# Plant growth-promoting bacteria as biological control agents for sustainable agriculture: targeting root-knot nematodes

**DOI:** 10.3389/fpls.2025.1567265

**Published:** 2025-08-19

**Authors:** Adriana S. Mata, Carlos Cruz, João Rodrigues Gaspar, Isabel Abrantes, Isabel Luci Conceição, Paula V. Morais, Diogo Neves Proença

**Affiliations:** ^1^ Centre for Mechanical Engineering, Materials and Processes (CEMMPRE), Advanced Production and Intelligent Systems (ARISE), Department of Life Sciences, University of Coimbra, Coimbra, Portugal; ^2^ Centre for Functional Ecology - Science for People and the Planet (CFE), Associate Laboratory TERRA, Department of Life Sciences, University of Coimbra, Coimbra, Portugal; ^3^ MED – Mediterranean Institute for Agriculture, Environment and Development & CHANGE – Global Change and Sustainability Institute, Faculdade de Ciências e Tecnologia, Universidade do Algarve, Faro, Portugal

**Keywords:** bacterial consortium, biological control agents, plant-parasitic nematodes, sustainable agriculture, *Bacillus amyloliquefaciens*, *Pseudomonas capeferrum*

## Abstract

The increasing frequency of extreme weather events affects ecosystems and threatens food production. The reduction of chemical pesticides, together with other ecological approaches, is crucial to more sustainable agriculture. Plant-parasitic nematodes (PPN), especially root-knot nematodes (RKN), *Meloidogyne* spp., are responsible for extensive damage to a wide range of economically important crops, leading to yield losses and reduced quality of the products. This study aims to show the potential of native potato-growing soil bacterial strains as biological control agents in a more sustainable agriculture perspective. After screening thirty bacterial strains, a bacterial consortium, composed of *B. amyloliquefaciens* UC_2.4, *P. capeferrum* UC_21.3 A.1, and *P. capeferrum* UC_21.30 A.1, was defined and investigated in more detail due to their potential for plant growth-promoting bacteria (PGPB), fungicidal, and nematicidal activities. The genomes of the strains were sequenced and analyzed for PGPB traits, and phenotypic assays were also performed. The nematicidal activity of these strains towards PPN and the model organism *Caenorhabditis elegans* was assessed. Their potential as PGPB and for controlling PPN on soil was evaluated in pot assays with tomato plants cv. Coração de Boi, by using bacterial strains alone and as a consortium. Here, the bacterial consortium showed some PGPB traits verified by genome mining and phenotypic assays *in vitro* and pot assays with plants. It was able to act as nematicidal agents with 100% efficacy towards PPN but not against *C. elegans*, indicating a highly targeted action mechanism, which might be attributed to the surfactin, fengycin, and lipopeptides, not affecting other non-target organisms that play essential roles in soil health. The bacterial consortium reduced the infectivity of PPN in plants by threefold. This bacterial consortium was established for the first time and has the potential to serve as a new tool for managing RKN in a more sustainable agricultural environment.

## Introduction

Climate change is one of the most important global challenges with huge implications for agriculture. The increasing frequency of extreme weather events affects ecosystems and threatens food production. To mitigate these impacts and ensure sustainable agricultural practices, it is vital that the use of chemical pesticides is reduced, and eco-friendly alternatives are explored (Varandas et al., 2020). Plant-parasitic nematodes (PPN) are a significant threat to crop production worldwide. They cause extensive damage to a wide range of economically important crops, leading to yield losses and reduced quality of the products (Pulavarty et al., 2021). Among PPN, root-knot nematodes (RKN), *Meloidogyne* spp., are the most damaging worldwide (Jones et al., 2013). This genus of sedentary endoparasites comprises more than 100 species parasitizing a wide range of hosts (Moens et al., 2009). Potato (*Solanum tuberosum*) is one of the most important crops in the world, being the fourth most consumed food, after rice, wheat, and corn. Rich in carbohydrates, fiber, vitamin C, potassium, and antioxidants, it is an energy base for millions of people. It is cultivated in more than 150 countries, and because it grows quickly, in different climates, and with good productivity, the potato is strategic in the fight against hunger. Furthermore, it generates billions of dollars in exports and jobs. One of the main causes of economic losses is the presence of PPN, mainly *Globodera* spp. and *Meloidogyne* spp (Mburu et al., 2020; Moens et al., 2021). Conventional methods of control of PPN face significant challenges that compromise their effectiveness and sustainability. The use of synthetic nematicides, although effective in the short term, presents serious environmental and public health problems. Furthermore, continued application of these products may lead to the development of resistance in nematodes. Biological methods, such as the use of fungi and bacteria, offer more environmentally friendly alternatives, but their effectiveness can be limited by a variety of factors (Bernard et al., 2017; Antil et al., 2023). Cultural practices, such as crop rotation and use of resistant cultivars, are also important strategies but face limitations. Crop rotation may be ineffective due to the wide host range of *Meloidogyne* spp., and genetic resistance may be rapidly overcome by nematodes, especially when there is constant selective pressure and exposure to high temperatures (Varandas et al., 2020). Therefore, changes are required, and innovative sustainable strategies should be developed to control PPN, such as the use of plant growth-promoting bacteria (PGPB) (Ramakrishna et al., 2019). These beneficial microbes have been shown to have wide traits that contribute to the health and growth of plants (Gunjal and Glick, 2024; Kumar et al., 2022), once they can solubilize essential nutrients like phosphate and zinc, making them more readily available to plants. They produce siderophores, which are chelating compounds that enhance the plant’s iron uptake by sequestering iron from the surrounding environment (Timofeeva et al., 2022a). Additionally, PGPB can express enzymes, including proteases and lipases, which are involved in the breakdown of proteins and lipids. Furthermore, some of these microbes are capable of producing indole acetic acid (IAA), a plant growth regulator that stimulates root development and increases the uptake of nutrients (Duca and Glick, 2020). PGPB may also have cellulolytic and chitinolytic activities that contribute to the decomposition of complex organic compounds, contributing to the recycling of organic matter in soil. Their catalase activity has been reported to help plants cope with oxidative stress, particularly under adverse environmental conditions (Timofeeva et al., 2022b). These characteristics and activities enable PGPB to improve plant growth, enhance nutrient availability, and increase resistance to various biotic and abiotic stressors, making them valuable for sustainable agriculture (Pardo-Díaz et al., 2021).

Bacterial strains belonging to the genera *Bacillus* and *Pseudomonas* have been recognized as PGPB, and some have been demonstrated to have the ability to control PPN effectively (Khabbaz et al., 2019). These strains exhibit multiple roles in agricultural ecosystems, serving as PGPB and acting as nematicides and/or fungicides. They can directly antagonize nematodes through the production of antimicrobial secondary metabolites and proteins and the formation of biofilms that physically inhibit nematode movement (Paiva et al., 2013; Proença et al., 2019a). Additionally, PGPB’s capacity to stimulate plant growth can indirectly enhance the host plant’s resistance to PPN infection by fortifying its natural defense mechanisms. These biological control agents are promising new control methodologies for sustainable agriculture by providing an eco-friendly means of managing PPN populations and reducing the amount of synthetic chemicals applied to soil (Raymaekers et al., 2020).

This study aims to show the potential of native potato-growing soil bacteria, *Bacillus* strain UC_2.4, and *Pseudomonas* strains UC_21.3 A.1 and UC_21.30 A.1 as biological control agents in a more sustainable agriculture perspective. The potential of bacterial strains as PGPB was assessed phenotypically and genomically, focusing on multiple key traits. The effects of the bacterial strains alone and, as the bacterial consortium, on plant growth were evaluated by their inoculation in pot assays with tomato plants. The nematicidal activity of these bacterial strains towards PPN, specifically *M. hapla* and *M. incognita*, as well as the model organism *Caenorhabditis elegans*, was evaluated. To assess the effectiveness of these strains in controlling PPN in the soil, an infectivity assay was conducted using *M. hapla* on tomato plants alongside the bacterial consortium.

## Materials and methods

### Bacterial consortium definition

Thirty bacterial strains were previously isolated from potato-growing soils in Portugal contaminated with PPN and deposited in the University of Coimbra Bacteria Culture Collection (UCCCB). Briefly, 5 g of soil were resuspended in 50 ml of sterile NaCl 0.85% (w/v), serial dilutions were obtained and plated in R2A agar medium (Difco Laboratories, Detroit, Michigan, USA), phosphate agar medium, and zinc agar medium, and incubated at 25°C for 5 days, according to Proença et al. (2019a, b). R2A was used for general heterotrophic bacteria isolation, and phosphate and zinc agar media were used to selectively isolate strains with nutrient solubilization abilities relevant to plant-growth promotion. Bacterial colonies from each soil sample were randomly isolated and preserved in LB medium (Difco Laboratories, Detroit, Michigan, USA) with 15% glycerol (v/v) stocks at −80°C after sub-cultivation and purification. The sampling sites are summarized in **Supplementary Table S1**. The microbial members of the consortium, composed of *Bacillus amyloliquefaciens* UC_2.4 (UCCCB 182), *Pseudomonas capeferrum* UC_21.3 A.1 (UCCCB 191), and *P. capeferrum* UC_21.30 A.1 (UCCCB 200), were selected based on complementary phenotypic PGPB traits and the fungicidal and nematicidal activities of each strain in preliminary tests through a PCA analysis (**Supplementary Figure S1**). The strains UC_21.3 A.1 and UC_21.30 A.1 were identified as belonging to the same species after the phylogenomic analysis mentioned below.

### Phylogenetic analysis

The strains were grown on R2A agar media at 25°C for 48h, and their genomic DNA was extracted using the NZY Microbial gDNA Isolation Kit (NZYTech, Lisbon, Portugal), according to the manufacturer’s instructions. The 16S rRNA gene was amplified by PCR and sequenced as previously described (Morais et al., 2004). The 16S rRNA gene sequences of all strains were then aligned with those of the type species of the closest genera and other reference sequences obtained from the EzTaxon-e server [http://eztaxon-e.ezbiocloud.net/; (Kim et al., 2012)] by SINA (v1.2.9), using the SILVA SEED as reference alignment [http://www.arb-silva.de/aligner/; (Pruesse et al., 2012)]. Sequences were included in the 16S rRNA-based Living Tree Project (LTP) release 128 databases by parsimony implemented in the ARB software package version 5.5 (Ludwig et al., 2004). Evolutionary distances were calculated (Jukes and Cantor, 1969), and phylogenetic dendrograms were constructed using the neighbor-joining (Saitou and Nei, 1987) and Randomized Axelerated Maximum Likelihood (RAxML) methods with the GTRGAMMA model (Stamatakis, 2006) included in the ARB software (Ludwig et al., 2004). Tree topologies were evaluated by performing bootstrap analysis (Felsenstein, 1985) of 1,000 datasets by using the ARB software package.

### Phylogenomics and genome mining

The sequencing libraries of strains UC_2.4, UC_21.3 A.1, and UC_21.30 A.1 were prepared according to the manufacturer’s instructions of the TruSeq Nano DNA (350 bp) High Throughput Library Prep Kit Illumina (TruSeq Nano DNA kit, NovaSeq platform), San Diego, California, USA. Briefly, 100 ng of genomic DNA was sheared using adaptive focused acoustic technology (Covaris, Woburn, Massachusetts, USA), and the fragmented DNA was end-repaired to create 5’-phosphorylated, blunt-ended dsDNA molecules. Following end-repair, DNA was size-selected with a bead-based method. These DNA fragments go through the addition of a single “A” base and ligation of the TruSeq DNA UD Indexing adapters. The products are then purified and enriched with PCR to create the final DNA library. The libraries were quantified using qPCR according to the qPCR Quantification Protocol Guide (KAPA Biosystems (KAPA Library Quantification kits), Wilmington, Massachusetts, USA) and qualified using the Agilent Technologies (4200 TapeStation D1000), Santa Clara, California, USA. After, the paired-end (2 × 150 bp) sequencing was performed by Macrogen using the NovaSeq Illumina (TruSeq Nano DNA kit, NovaSeq platform), San Diego, California, USA. The reads obtained were analyzed by applying FastQC and TrimGalore, including the Phred20 quality filter by Macrogen, Seoul, South Korea. The genome assembly was performed by using SPAdes 3.5 (Prjibelski et al., 2020), included in Unicycler v0.5.0 (Wick et al., 2017). The assembled genomes were annotated by using automatic pipelines of NCBI Prokaryotic Genomes Automatic Annotation Pipeline (PGAP, http://www.ncbi.nlm.nih.gov/genomes/static/Pipeline.html) and RAST (Aziz et al., 2008).

The genome sequences of strains UC_2.4, UC_21.3 A.1, and UC_21.30 A.1 were also analyzed by using the type strain genome server (TYGS), including all dependencies in the webtool, for additional phylogenomic analysis (Meier-Kolthoff et al., 2022; Meier-Kolthoff and Göker, 2019). Genome distances were determined by calculating the digital DNA–DNA hybridization (DDH) (Meier-Kolthoff et al., 2022; Meier-Kolthoff and Göker, 2019). The G+C content of the genome was determined based on the genome sequence of all three strains.

All annotated draft genomes were subjected to a search for genes encoding proteins recognized as relevant in plant growth promotion processes by using Rapid Annotation using the Subsystem Technology (RAST) server. These genes included 1-aminocyclopropane-1-carboxylic acid (ACC) deaminase (WP_009638971), leucine-responsive regulatory protein/asparagine synthase C (Lrp/AsnC) products (WP_009638972), cellulase-β-glucosidase (WP_009635056, WP_009636821), periplasmic β-glucosidase (WP_009638496), catalase (WP_009638086), catalase/peroxidase (WP_009636268), pectinase-pectinesterase B (WP_009638872), superoxide dismutase (SOD, WP_009635546), chitinase (WP_009638226), polysaccharide deacetylase (*nodB*; WP_009639040, WP_009638391, WP_009639041), nitrogenase (*nifH*; AUG99286.1, H650_03210 K02588, pRL100162 K02588), aromatic L-amino acid decarboxylase (WP_009635138), aliphatic amidase AmiE (WP_009634947, WP_009639043), nitrilase (WP_009634944), aldehyde dehydrogenase (WP_009635369, WP_009635627, WP_009636235, WP_009637232, WP_009639021, WP_009639329), pyruvate dehydrogenase (WP_009634667), acetolactate synthase (WP_009635236, WP_009634704, WP_009634705, WP_009637883, WP_009637884), alpha-acetolactate decarboxylase (WP_009635235), 2,3-butanediol dehydrogenase (WP_009635231), acyl-homoserine lactones (AHLs) synthase (WP_009638744, WP_009635293), LuxR family transcriptional regulator (WP_009638745, WP_009635292, WP_009637809, WP_009638756, WP_009635139, WP_037377293), AsnC family leucine-responsive regulatory protein (WP_009638972), D-cysteine desulfhydrase (WP_009636121), myrosinase (NP_001302796.1, ALM58466.1), and hydrocyanic acid (HCN). All annotated draft genomes were subjected to secondary biosynthetic gene cluster analysis by using the platform antiSMASH 8.0 (Blin et al., 2025).

### *In vitro* characterization of bacterial isolates

A deeper characterization of each bacterial consortium member was carried out in multiple *in vitro* tests to assess the PGPB potential and the nematicidal effect against *M. hapla* (AM 4) and the fungicidal activity against *Fusarium oxysporum* L21A50–1 and *Botrytis cinerea* Pars. The phosphate and zinc solubilization, siderophore production, protease and lipase activities, IAA production, cellulolytic and chitinolytic activities, catalase activity, and nematicidal and fungicidal activities were performed according to Proença et al. (2019a, b).

### Antibiotic susceptibility testing of bacterial consortium

The bacterial strains were subjected to antimicrobial susceptibility testing using the disk diffusion method, according to the guidelines of the European Committee on Antimicrobial Susceptibility Testing (EUCAST) (https://www.eucast.org/). The inoculum consisted of suspended colonies in a sterile saline solution adjusted to a 0.5 McFarland turbidity standard. After the inoculation on Mueller-Hinton agar (Oxoid (Mueller-Hinton agar), Basingstoke, Hampshire, United Kingdom) plates, the 6-mm antibiotic disks were applied to the surface of the inoculated agar plate and incubated at 35 ± 1°C for 16h–20h. The results were interpreted as sensitive, intermediate, or resistant according to the inhibitory zone diameters around the disks using EUCAST breakpoint tables (EUCAST, 2024). The antibiotic disks included oxacillin (1 µg), ampicillin (10 µg), ceftriaxone (30 µg), kanamycin (30 µg), neomycin (30 µg), meropenem (10 µg), erythromycin (15 µg), novobiocin (30 µg), tetracycline (30 µg), eifampin (30 µg), chloramphenicol (30 µg), and polymyxin B (300 µg).

### Maintenance and multiplication of the root-knot nematode population

*Meloidogyne hapla* (AM 4) and *M. incognita* (AM 31) populations, sourced from the NEMATO-lab collection of the University of Coimbra, were selected because they are two of the most common RKN species worldwide and have a broad host range of significant economic importance (Rusinque et al., 2022). Tomato plants, *Solanum lycopersicum* cv. Coração de Boi, were grown from seeds germinated at 25°C–27°C for approximately 3 days, in the dark, on moist filter paper in Petri dishes, and transplanted singly into 5-cm diameter plastic pots, containing 60 cm^3^ of a steam-sterilized mixture of loam soil and sand-peat (1:2, w/w) as follows: 68.0% thin ground (Ø < 2 mm), 1.2% organic matter content, 53 mg P_2_O_5_ kg^−1^ extractable phosphorus, 24 mg K_2_O kg^−1^ extractable potassium, and pH = 6.2. Granulometric analysis: 1.2 organic matter, 89.08 coarse sand, 5.48 thin sand, 3.21 slime, 2.23 clay (Robinson’s pipette method (Silva, 1975), and the texture class is sandy. The plants were kept in a greenhouse (20°C–25°C, 70%–75% relative humidity, and 12h photoperiod).

*Meloidogyne* populations were maintained and multiplied in 3- to 4-week-old tomato plants inoculated with 10–15 egg masses each, or with 5,000 second-stage juveniles (J2)/plant, extracted from infected roots, using 0.5% sodium hypochlorite (NaOCl) according to the methodology described by Hussey and Barker (1973). The plants were kept in a greenhouse under the same conditions as described above. Sixty days after inoculation (DAI), the tomato plants were uprooted, the egg masses collected from the roots, and new plants inoculated. Esterase phenotype was used to confirm the species before and after each assay (Maleita et al., 2012; Pais and Abrantes, 1989).

The nematode wild type of *Caenorhabditis elegans* N2 was maintained on agar plates containing *Escherichia coli* OP50 at 19.5°C (Proença et al., 2019a). Briefly, this wild type, obtained from the Caenorhabditis Genetic Center, Minneapolis, Minnesota, USA, was propagated at 19.5°C on agar medium (NGM) with *E. coli* OP50 lawn (Brenner, 1974). After 3 days, nematodes were harvested by rinsing the plates with sterile M9 buffer (3 g KH_2_PO_4_, 6 g Na_2_HPO_4_, 5 g NaCl, 1 ml 1M MgSO_4_, dH_2_O to 1 L) and transferred to 15 ml tubes in a total of 3.5 ml. A fresh solution was added containing 0.5 ml of 2N NaOH and 1 ml of 2% sodium hypochlorite, followed by vortexing every 2 min with a final duration of 10 min. The nematodes and eggs were centrifuged at 1,300*g*, 30 s at 16°C. Supernatant was removed, and the nematodes and eggs were washed two times with sterile distilled water (SDW). The nematodes and eggs were then transferred into new NGM plates with *E. coli* OP50 lawn and incubated at 19.5°C for 18h–24h.

### Effect of bacterial consortium on plant germination and growth: from *in vitro* to pot assays

To determine the effect of the bacterial strains on germination, seedling vigor and plant growth (root and shoot length), three different media were tested, namely, CAA, LB medium, and LB medium supplemented with 500 µg/ml tryptophan (LB + Trp). Tomato seeds of cv. Coração de Boi were surface sterilized in 2% sodium hypochlorite (NaOCl) for 5 min and washed three times with SDW. Sterilized seeds were immersed for 30 min in each bacterial strain inoculum (OD_600_ = 0.6, at a concentration of 1 × 10^8^ CFU.ml^−1^) and in a suspension of the bacterial consortium containing all three bacterial strains. As control treatments, SDW and non-inoculated media were used. Ten inoculated seeds were placed on a water-agar (0.25%, w/v) plate for each treatment and incubated for seven days. All assays were performed across two independent experiments. Germination rate and plant growth measurements (root and shoot length) were analyzed after the seventh day. The vigor index was calculated using the following formulas.


$$\text{Germination rate }\left(\%\right)=\left(\frac{\text{Number of seeds germinated}}{\text{Total number of seeds}}\right)\times 100$$

Vigor index =%Germination ×Total plant length

In the pot experiments, a bacterial consortium (UC_2.4, UC_21.3A.1 and UC_21.30A.1) was tested for plant growth-promotion on tomato plants cv. Coração de Boi. Tomato seeds were sterilized with 2% NaOCl, washed with SDW to remove any residual NaOCl, and allowed to germinate at 25°C for 3 days. The germinated seeds were then transferred to sterilized plastic pots (5.7 cm diameter, ca. 47.9 g soil) filled with a sterilized mixture of sand-peat (1:2, w/w) soil (Siro-germ substrate and river sand, Le Roy Merlin (Siro-germ substrate & supplies), Lille, France) as follow: 0–8 mm granulometry, >70% organic matter content, pH = 5.0**–**6.0, 150–200 µs/cm conductivity, composition of Siro Agro 1 (pine bark humus—RAL certified), sphagnum blonde peat, coco peat and generic mineral fertilizer start; mineral fertilization of NPK 13-0-13: 1 kg/m^3^. Each bacterial strain of the consortium, grown individually in three different media (LB + Trp, CAA, and LB medium), at 26°C, in a shaker incubator (160 rpm) for 24h, was diluted with the respective culture medium to an OD_600_ = 0.6 (≈ 1.0 × 10^8^ CFU.ml^−1^ concentration). At the time of potting, plants were inoculated with six different treatments for each culture medium (1 ml), including two controls (SDW and non-inoculated medium), the three individuals bacterial inocula of the consortium and a suspension containing all bacterial inocula. All the tests were performed with three replicates, and two treatments were tested: single exposure only inoculated at the time of potting and several inoculations (one/week) till the end of the assay. Four weeks after the first treatment, the tomato plants were uprooted, measured and weighed to obtain the plant growth parameters of shoot and root.

### Nematicidal activity of bacterial supernatants against the plant-parasitic nematodes *M. hapla*, *M. incognita*, and the model nematode *C. elegans*

The isolated bacterial strains were evaluated for *in vitro* nematicidal activity. The strains were grown in three culture media as described above at 26°C, in a shaker incubator (160 rpm) for 24h. Bacterial cultures were centrifuged (15 min, 4°C, 17,000*g*), and the resulting supernatants were filtered through a 0.22 µm sterile syringe filter. Then, 500 µl of each sterilized culture filtrate was transferred to each well of a 24-well cell culture plate, containing 50–70 disinfected J2 of *M. hapla*. Nematodes were disinfected by sequential washes in chlorhexidine digluconate 0.2% (one wash, 1 min) and in SDW (three washes, 1 min/each). Distilled water and medium were used as controls, and all treatments were conducted with five replicates. After 24h of exposure, mortality was determined by counting the number of dead and live *M. hapla* J2 under a low-power stereomicroscope. Nematodes were considered dead when straight and immobile and not able to recover after being transferred to water. *Meloidogyne incognita* was also tested, as mentioned for *M. hapla*, to evaluate the efficacy of the nematicidal activity of non-diluted bacterial supernatants obtained from bacterial growth in CAA medium.

The nematicidal activity of bacterial supernatants was evaluated towards *C. elegans* N2 (mixed life stages: L4 and adults), according to Proença et al. (2019a). The bacterial suspensions were centrifuged (15 min, 4°C, 17,000*g*), and the resulting supernatants were filtered through a 0.22 µm sterile syringe filter. To test the nematicidal activity, 500 μl of each bacterial filtered supernatant was incubated with 20 disinfected nematodes for 24h at 20°C. Five replicates for each treatment were done. Nematodes were disinfected by sequential washes in 0.1% sodium hypochlorite (one wash, 1 min at RT) and 1 ml sterilized washing buffer, like M9 buffer (two washes, 1 min at RT), followed by centrifugation. The last wash (100 μl) was inoculated on LB agar for control of the disinfection efficiency. The number of dead nematodes was calculated under a low-power stereomicroscope. Nematodes were considered dead, as mentioned above. The controls, nematodes in the corresponding medium, were incubated under the same conditions.

### Infectivity of *M. hapla*

Tomato plants, cv. Coração de Boi, were used to evaluate the nematode’s infectivity. The assay, in pots (7 cm diameter, ca. 60 g soil), one plant per pot, was prepared as previously described for PGPB. The treatments included: the control, plants without the bacterial consortium and inoculated with 300 J2 of *M. hapla*, 5 nematodes/g soil (Collett et al., 2024); plants inoculated with bacterial consortium (1 ml of each strain, adjusted to OD_600_ = 0.6 and equivalent to ≈ 1.0 × 10^8^ CFU.ml^−1^) and, after 30 min, inoculated with 300 J2 of *M. hapla*; and plants inoculated with 300 J2 of *M. hapla* and, after 30 min, inoculated with the bacterial consortium. Each treatment was conducted with five replicates. After 1 week, the plants were uprooted and, after being carefully washed out of debris, the roots were stained with acid fuchsin (Bybd et al., 1983). The RKN infectivity was determined by counting the number of stained *M. hapla* nematodes inside the roots under a low-power stereomicroscope.

### Data analyses

Data analyses were performed using the RStudio software. All plots were created using the ggplot2 and ggpubr packages. The rstatix and R base packages were used for the statistical analysis of all plots. The differences between all treatments were evaluated through one-way (infectivity assays), two-way (nematicidal activity with *C. elegans* and germination assays) and three-way (nematicidal activity with *M. hapla* and pot experiments) ANOVA, followed by Tukey’s pairwise comparisons. The insertion of statistical analysis results into the plots was performed using the multcomp and multcompView packages. The level of statistical significance for all analyses was α = 0.05.

### Nucleotide sequence accession numbers

The 16S rRNA gene sequences of the bacterial isolates reported in this study were deposited at the GenBank database under the accession numbers PP825709–PP825738.

This Whole Genome Shotgun project of strains UC_2.4, UC_21.3 A.1, and UC_21.30 A.1 has been deposited at DDBJ/ENA/GenBank under the accessions JAWWVB000000000, JAWWVA000000000, JAWWUZ000000000. The version described in this paper is version JAWWVB010000000, JAWWVA010000000, JAWWUZ010000000, respectively.

## Results

### Phylogenomics and genome mining

Phylogenetic analyses of the 16S rRNA gene sequences of isolated strains with those of the type species of all recognized species of all the related genera were performed. According to the maximum-likelihood and neighbor-joining phylogenetic trees, the strains were identified at the genus level (**Supplementary Table S1**), except for the three bacterial strains of the consortium that were identified at the species level after the complete analysis described below.

**Figure 1:**
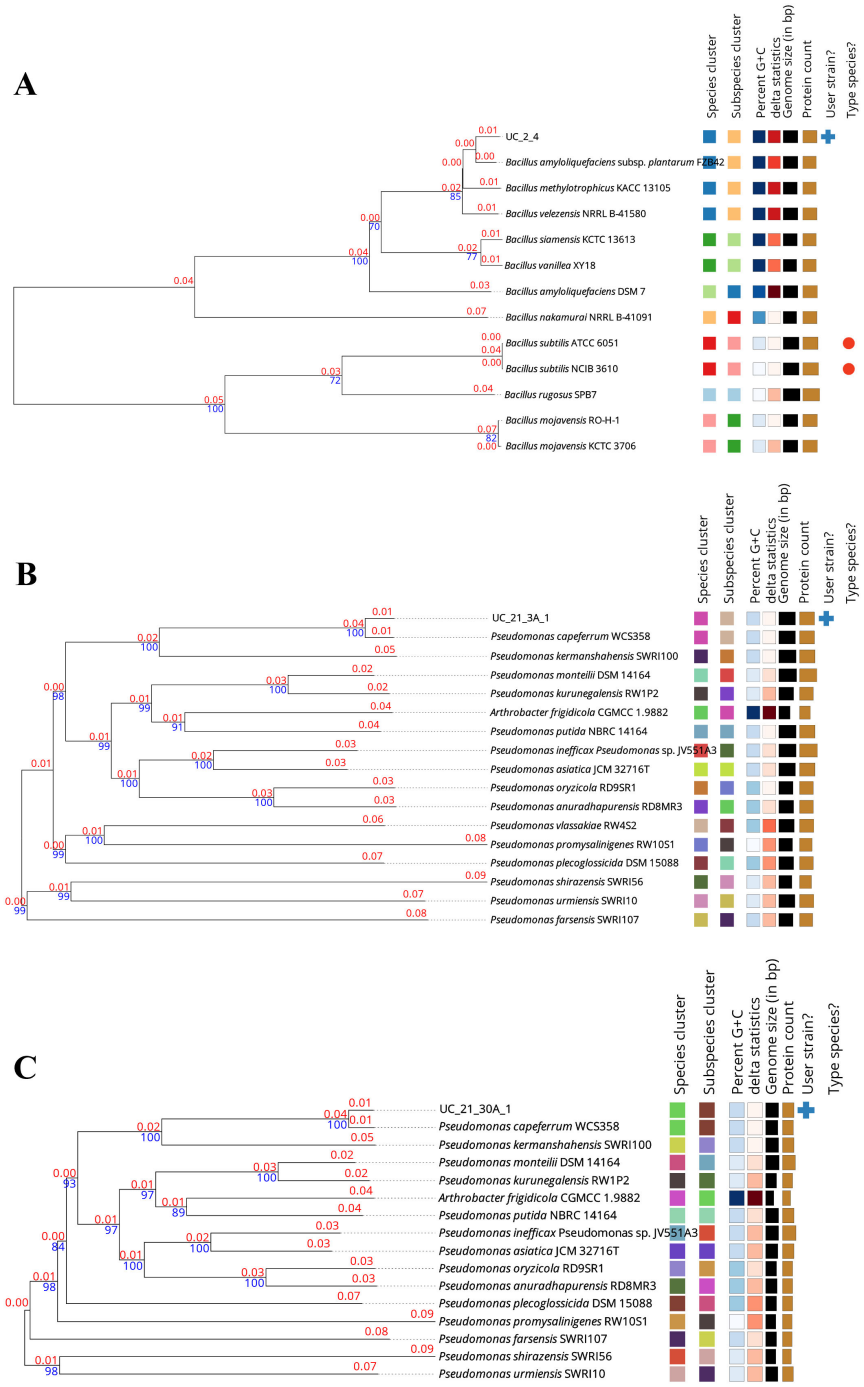
Phylogenomic trees of the bacterial strains (UC_2.4, UC_21.3 A.1 and UC_21.30 A.1) used as the bacterial consortium in this work. The trees are based on Genome BLAST Distance Phylogeny (GBDP) distances calculated from genome sequences using the Type (Strain) Genome Server (TYGS). The strains UC_2.4 **(A)**, UC_21.3 A.1 **(B)**, and UC_21.30 A.1 **(C)** were identified as *Bacillus amyloliquefaciens*, *Pseudomonas capeferrum*, and *P. capeferrum*, respectively. The blue numbers on the tree indicate the percentages of bootstrap sampling, derived from 1,000 replications; and the red numbers on the tree indicate the branch lengths scaled in terms of the used GBDP distance formula.

The phylogenomic trees of each bacterial strain that composes the consortium used in this work, based on GBDP distances calculated from genome sequences through the TYGS server, showed that strains UC_2.4, UC_21.3 A.1, and UC_21.30 A.1 belong to the species *Bacillus amyloliquefaciens*, *Pseudomonas capeferrum*, and *P. capeferrum*, respectively (**Figure 1**). The analyses of DDH between UC_2.4, UC_21.3 A.1, and UC_21.30 A.1, and their closest relatives by using the available sequences from the TYGS online tool are summarized in **Table 1**.

**Table 1:** Pairwise comparisons of genomes of strains UC_2.4, UC_21.3 A.1, and UC_21.30 A.1 versus type-strain genomes available in TYGS database.

Query strain	Subject strain	dDDH (d4, in %)	C.I. (d4, in %)	G + C content difference (in %)
UC_2.4	*Bacillus amyloliquefaciens* subsp. *plantarum* FZB42	91.2	[89.1–93.0]	0.02
UC_2.4	*Bacillus methylotrophicus* KACC 13105	84.5	[81.7–86.9]	0.03
UC_2.4	*Bacillus velezensis* NRRL B-41580	84.4	[81.6–86.8]	0.14
UC_2.4	*Bacillus siamensis* KCTC 13613	56.9	[54.1–59.6]	0.12
UC_21.3 A.1	*Pseudomonas capeferrum* WCS358	90	[87.6–91.9]	0.05
UC_21.3 A.1	*Pseudomonas kermanshahensis* SWRI100	41.4	[38.9–43.9]	0.4
UC_21.3 A.1	*Pseudomonas asiatica* JCM 32716^T^	34.4	[31.9–36.9]	0.08
UC_21.3 A.1	*Pseudomonas inefficax* JV551A3	34.1	[31.7–36.6]	0.21
UC_21.30 A.1	*Pseudomonas capeferrum* WCS358	90.4	[88.1–92.3]	0.09
UC_21.30 A.1	*Pseudomonas kermanshahensis* SWRI100	41.4	[38.9–44.0]	0.36
UC_21.30 A.1	*Pseudomonas asiatica* JCM 32716^T^	34.4	[32.0–36.9]	0.04
UC_21.30 A.1	*Pseudomonas inefficax* JV551A3	34.1	[31.7–36.6]	0.25

The general features of draft genome sequences of strains UC_2.4, UC_21.3 A.1, and UC_21.30 A.1 are summarized in **Table 2**. The genomes of strains UC_2.4, UC_21.3 A.1, and UC_21.30 A.1 were assembled into 24, 67, and 48 contigs with more than 500 bp, totaling 3,917,685 bp, 5,904,689 bp, and 6,004,693 bp with a mapped coverage of 385.1-, 271.2-, and 252.2-fold of the genome, respectively. The G + C content of the DNA was 46.46%, 62.62%, and 62.58%, respectively. The genomes encoded a total of 3,857, 5,367, and 5,422 genes and 3,798, 5,299, and 5,356 putative coding sequences (CDSs, with protein), respectively.

**Table 2:** General genome features of *Bacillus amyloliquefaciens* UC_2.4, *Pseudomonas capeferrum* UC_21.3 A.1, and *Pseudomonas capeferrum* UC_21.30 A.1.

STRAIN	UC_2.4	UC_21.3 A.1	UC_21.30 A.1
BioProject	PRJNA1037594	PRJNA1037594	PRJNA1037594
BioSample	SAMN38191810	SAMN38191811	SAMN38191812
Accession	JAWWVB000000000	JAWWVA000000000	JAWWUZ000000000
Genome Coverage	385.1×	271.2×	252.2×
Genome size (bp)	3,917,685	5,904,689	6,004,693
G + C %	46.46	62.62	62.58
Genes (total)	3,857	5,367	5,422
CDSs (total)	3,798	5,299	5,356
Genes (coding)	3,731	5,227	5,284
CDSs (with protein)	3,731	5,227	5,284
Genes (RNA)	59	68	66
rRNAs	1, 1, 1 (5S, 16S, 23S)	2, 1, 3 (5S, 16S, 23S)	2, 1, 4 (5S, 16S, 23S)
completerRNAs	1, 1, 1 (5S, 16S, 23S)	2 (5S)	2, 1 (5S, 16S)
partialrRNAs	–	1, 3 (16S, 23S)	4 (23S)
tRNAs	51	58	55
ncRNAs	5	4	4
PseudoGenes (total)	67	72	72
CDSs (without protein)	67	72	72
PseudoGenes (ambiguous residues)	0 of 67	0 of 72	0 of 72
PseudoGenes (frame shifted)	36 of 67	25 of 72	27 of 72
PseudoGenes (incomplete)	42 of 67	64 of 72	60 of 72
PseudoGenes (internal stop)	7 of 67	3 of 72	6 of 72
PseudoGenes (multiple problems)	17 of 67	18 of 72	19 of 72

**Figure 2:**
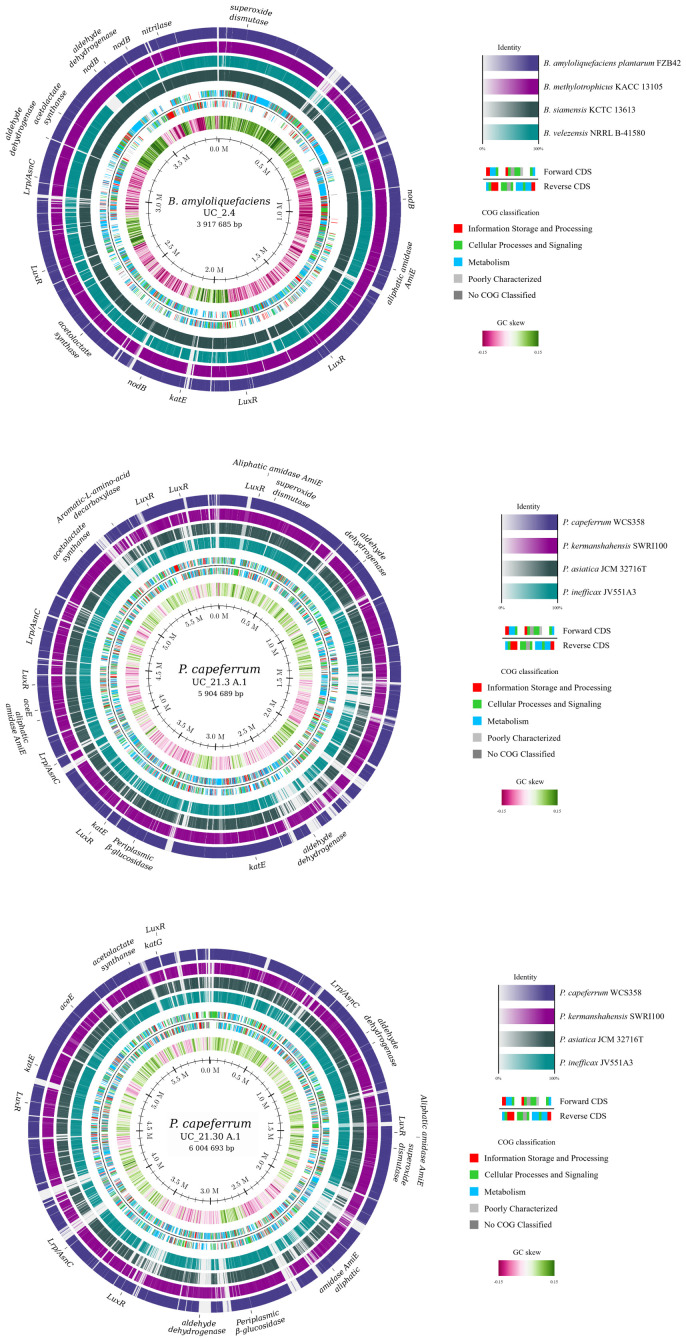
Genome map of the bacterial consortium strains (UC_2.4, UC_21.3 A.1, and UC_21.30 A.1) using the genome mining in RAST server and highlighting numerous genes encoding proteins recognized as relevant in plant growth-promoting processes. These genes encoding for leucine-responsive regulatory protein/asparagine synthase C (Lrp/AsnC) products, periplasmic β-glucosidase, catalase, catalase/peroxidase, superoxide dismutase (SOD), polysaccharide deacetylase (NodB), aromatic L-amino acid decarboxylase, aliphatic amidase AmiE, nitrilase, aldehyde dehydrogenase, pyruvate dehydrogenase, acetolactate synthase, alpha-acetolactate decarboxylase, LuxR family transcriptional regulator, and AsnC family leucine-responsive regulatory protein.

The genome mining of the bacterial consortium was performed using the RAST server to analyze numerous genes encoding proteins recognized as relevant in plant growth-promoting processes (**Figure 2**; **Supplementary Table S4**). Strains UC_21.3 A.1 and UC_21.30 A.1 exhibited a high degree of similarity, sharing the same profile in all the genes examined related to plant growth promotion. However, 212 and 481 genes were found as unique genes of strains UC_21.30 A.1 (**Supplementary Table S4**) and UC_21.3 A.1 (**Supplementary Table S4**), respectively. From that, 73.6% and 50.7% of the genes were identified as encoding for hypothetical proteins, respectively.

When comparing the *Pseudomonas* strains, strain UC_21.30 A.1 has genes coding for the azotobactin D-like siderophore not present in strain UC_21.3 A.1. The genome of *Bacillus* strain UC_2.4 showed the presence of biosynthetic gene clusters for fengycin, surfactin, and the metallophore bacillibactin (**Supplementary Figure S2**). The phenylalanine, tyrosine, and tryptophan biosynthetic pathway was represented in both strains, with 67.7% in UC_21.30 A.1. Both strains possess genes for AsnC family leucine-responsive regulatory protein (Lrp), periplasmic beta-glucosidase, catalase, catalase/peroxidase, SOD, aromatic-L-amino-acid decarboxylase, aliphatic amidase AmiE, aldehyde dehydrogenase, pyruvate dehydrogenase, acetolactate synthase, and LuxR family transcriptional regulator. However, UC_2.4 differs in some genes from UC_21.3 A.1 and UC_21.30 A.1. Strain UC_2.4 possessed genes for nodB, nitrilase, and alpha-acetolactate decarboxylase, which were absent in the other two strains. Additionally, unlike UC_21.3 A.1 and UC_21.30 A.1, strain UC_2.4 lacked genes for periplasmic beta-glucosidase, catalase/peroxidase, aromatic L-amino acid decarboxylase, and pyruvate dehydrogenase.

### *In vitro* characterization of bacterial isolates

In this study, the bacterial consortium consisting of three strains, *B. amyloliquefaciens* UC_2.4, *P. capeferrum* UC_21.3 A.1, and *P. capeferrum* UC_21.30 A.1, was screened for various PGPB properties, antibiotic susceptibility, fungicidal, and nematicidal activities. Both strains, UC_21.3 A.1 and UC_21.30 A.1, exhibited similar characteristics in terms of zinc solubilization, siderophore production, and catalase activity (**Supplementary Table S1**). Strain UC_21.3 A.1 demonstrated weak phosphate solubilization and produced 116 µg ml^−1^ of IAA, with fungicidal activity only against *B. cinerea*. In contrast, strain UC_21.30 A.1 showed no phosphate solubilization, produced 481 µg ml^−1^ IAA, and also had fungicidal activity against *B. cinerea*. On the other hand, strain UC_2.4 stood out with its phosphate solubilization, protease production, and cellulolytic activity. It shared siderophore and IAA production and catalase activity with the other strains but lacked zinc solubilization, lipase production, and chitinolytic activity. Moreover, strain UC_2.4 exhibited fungicidal activity against *Fusarium oxysporum* but was ineffective against *B. cinerea*. All strains showed 100% nematicidal activity towards *M. hapla* (**Supplementary Table S1**).

### Antibiotic susceptibility testing of bacterial consortium

**Figure 3:**
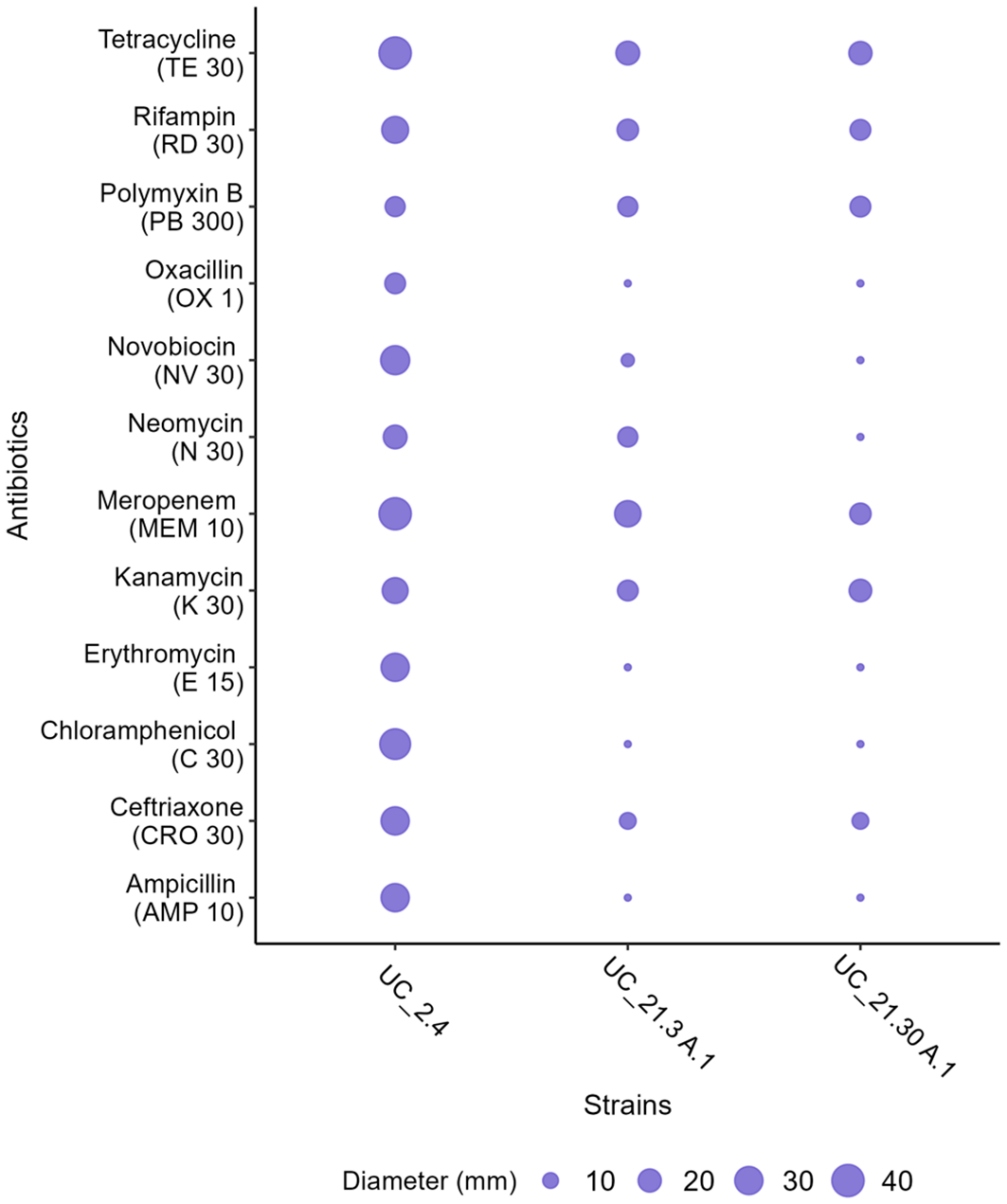
Antibiotic susceptibility test of the bacterial consortium strains (*B. amyloliquefaciens* UC_2.4, *P. capeferrum* UC_21.3 A.1, and *P. capeferrum* UC_21.30 A.1) using the EUCAST disk diffusion method. The zone diameters (in millimeters) were measured for the following antibiotics: oxacillin (1 µg), ampicillin (10 µg), ceftriaxone (30 µg), kanamycin (30 µg), neomycin (30 µg), meropenem (10 µg), erythromycin (15 µg), novobiocin (30 µg), tetracycline (30 µg), rifampicin (30 µg), chloramphenicol (30 µg), and polymyxin B (300 µg).

In terms of antibiotic susceptibility, *B. amyloliquefaciens* UC_2.4 showed more susceptibility to the antibiotics tested compared with the other two strains. This is evidenced by comparing the zone diameters of the bacterial strains in **Figure 3** (**Supplementary Figure S3**; **Supplementary Table S5**). This heightened susceptibility was particularly notable for meropenem. When comparing strains, strain UC_2.4 was more susceptible to erythromycin than *Pseudomonas* strains. Moreover, *P. capeferrum* UC_21.3 A.1 and *P. capeferrum* UC_21.30 A.1 showed similar antibiotic reactions, except for UC_21.30 A.1, which exhibited resistance to meropenem and differences in zone diameters regarding the neomycin test (**Figure 3**; **Supplementary Table S5**). We also found differences in oxacillin, novobiocin, chloramphenicol, and ampicillin when comparing UC_2.4 with the other two strains. However, no records of resistance to these antibiotics were found for *Bacillus* spp.

### Nematicidal activity of bacterial supernatants against the plant-parasitic nematode *M. hapla*, *M. incognita*, and the free-living nematode *C. elegans*

**Figure 4:**
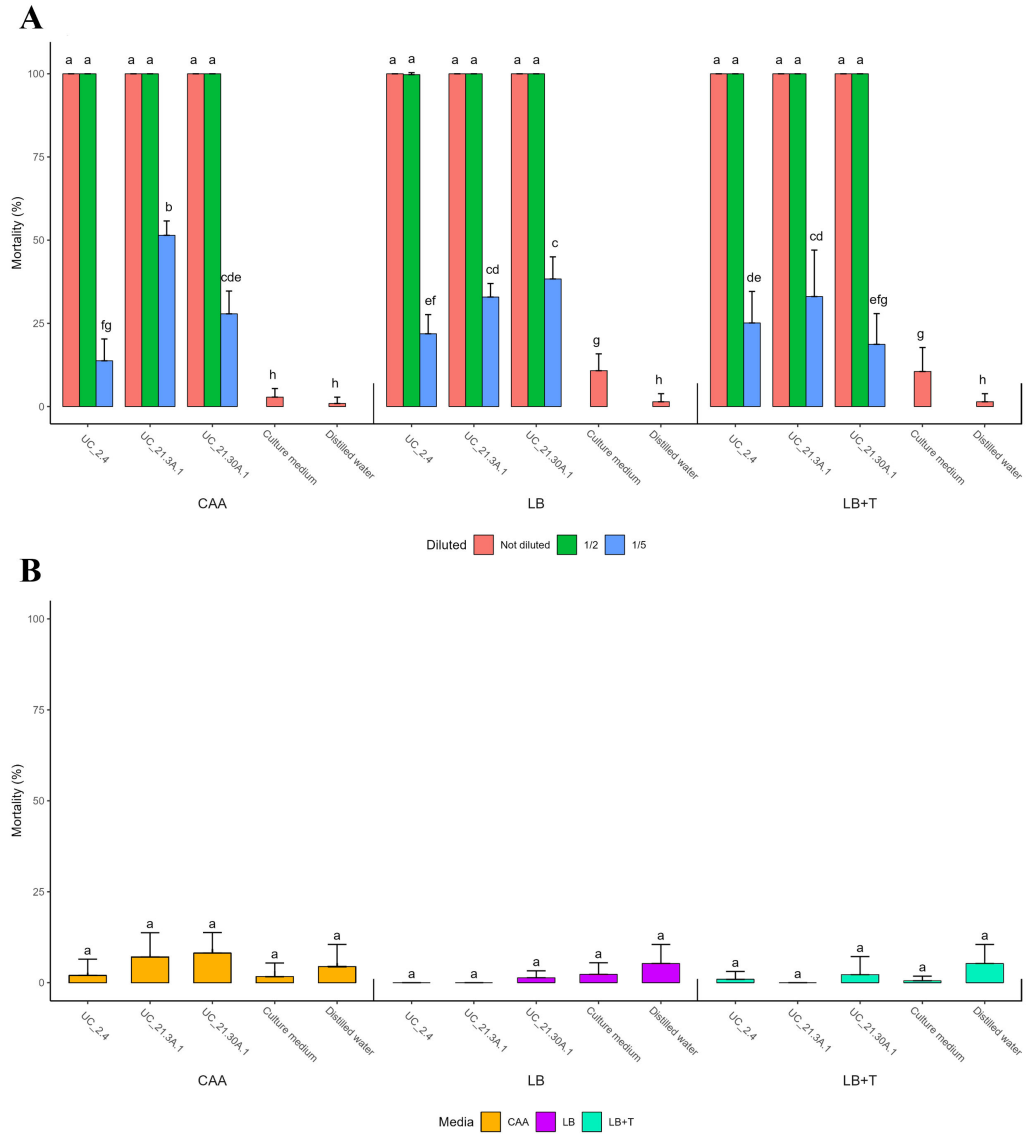
Nematicidal activity of bacterial supernatants towards RKN and *C*. *elegans*. Filtered bacterial supernatants (diluted and non-diluted) from the bacterial consortium strains (*B. amyloliquefaciens* UC_2.4, *P. capeferrum* UC_21.3 A.1, and *P. capeferrum* UC_21.30 A.1) grown in CAA, LB and LB supplemented with 500 μg/ml tryptophan (LB + Trp) media were tested towards the plant-parasitic nematode *Meloidogyne hapla* **(A)** and the free-living nematode *Caenorhabditis elegans* **(B)**. Five replicates per treatment. Standard deviations indicate variability among replicates. Different letters showing statistical differences (*p* < 0.05).

Supernatants from filtered bacterial consortium growths (diluted and not diluted) in CAA, LB, and LB + Trp media were used to evaluate *in vitro* nematicidal activity against *M. hapla* AM4 (**Figure 4A**), *M. incognita* AM31 (**Supplementary Figure S4**), and *C. elegans* N2 (**Figure 4B**). Non-diluted and 50% diluted bacterial supernatants obtained from growth in all tested culture media showed 100% nematicidal activity towards *M. hapla* AM 4 (**Figure 4A**).

Moreover, all bacterial supernatants showed nematicidal activity with statistical significance on *M. hapla* AM4 compared to their respective controls, except for strain UC_21.30 A.1 20% diluted in LB + Trp medium. Nematicidal activity against *C. elegans* N2 was conducted under the same conditions but only using non-diluted bacterial supernatants. Bacterial supernatants from all tested culture media did not show significant nematicidal activity against *C. elegans* N2, with the highest average mortality rate of 8.1% (**Figure 4B**). The diluted supernatants were not tested on *C. elegans* N2 due to the low nematicidal activity of non-diluted supernatants.

### Effect of bacterial consortium on plant germination and growth: from *in vitro* to pot assays

**Figure 5:**
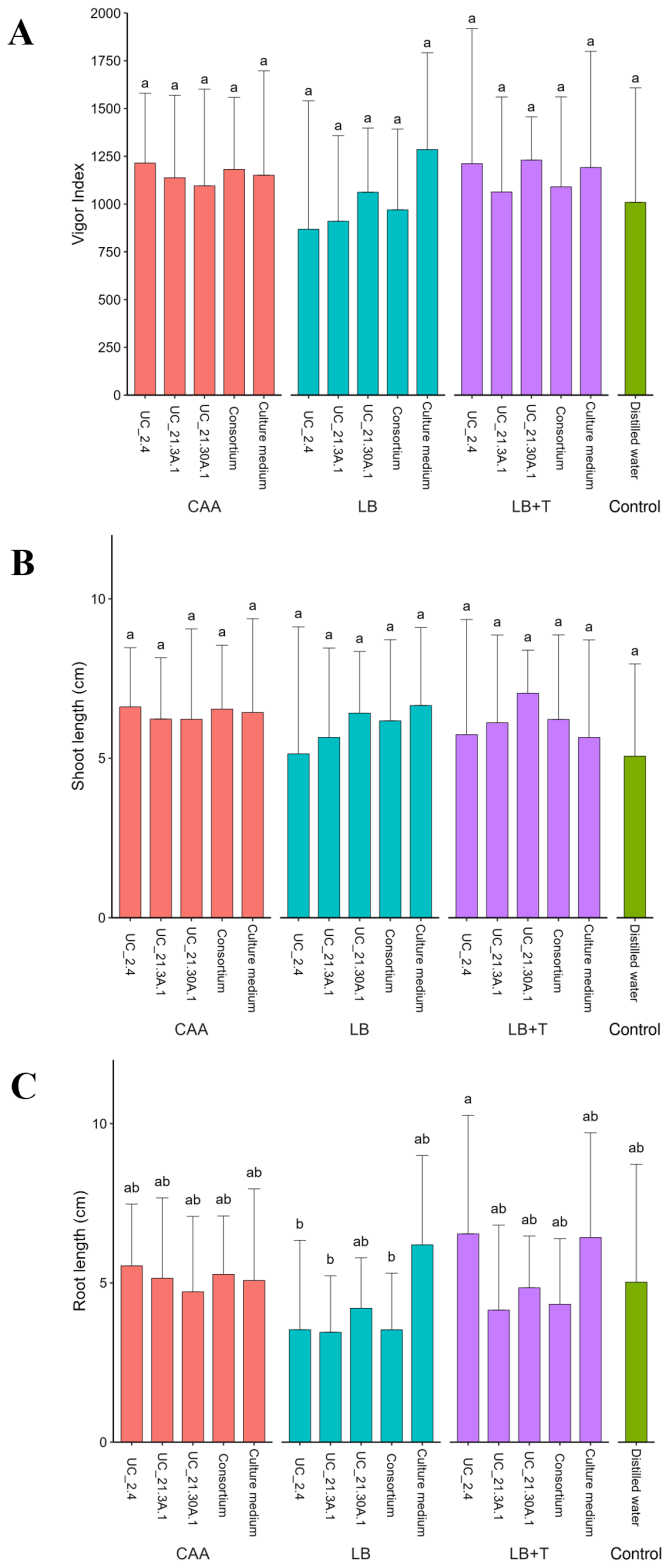
Effect of bacterial consortium strains on plant germination (**A**, vigor index) and *in vitro* growth (**B**, **C**, shoot and root length in centimeters, respectively) in tomato plants across different bacterial growth media (CAA, LB, LB + Trp). Tomato seeds (*S. lycopersicum* cv. Coração de Boi) were treated with individual or consortium strains, placed on a water-agar (0.25%, w/v) plate and incubated for seven days. High standard deviations indicate variability among replicates. Twenty replicates per treatment. Different letters showing statistical differences (*p* < 0.05).

The different media did not affect the vigor index and the shoot length of the tomato plants, although the variation was high between replicates, as can be seen by the high standard deviations (**Figure 5**). The treatments with the consortium did not present differences compared to the control. The treatments with bacterial growth in CAA showed no variation in the root length, but some differences were observed when bacterial growth was performed in LB and LB + Trp media. The root treated with strain UC_2.4 with LB + Trp medium showed a higher length with statistical relevance compared with strains UC_2.4, UC_21.3A.1, and the bacterial consortium incubated in LB medium (**Figure 5**).

**Figure 6:**
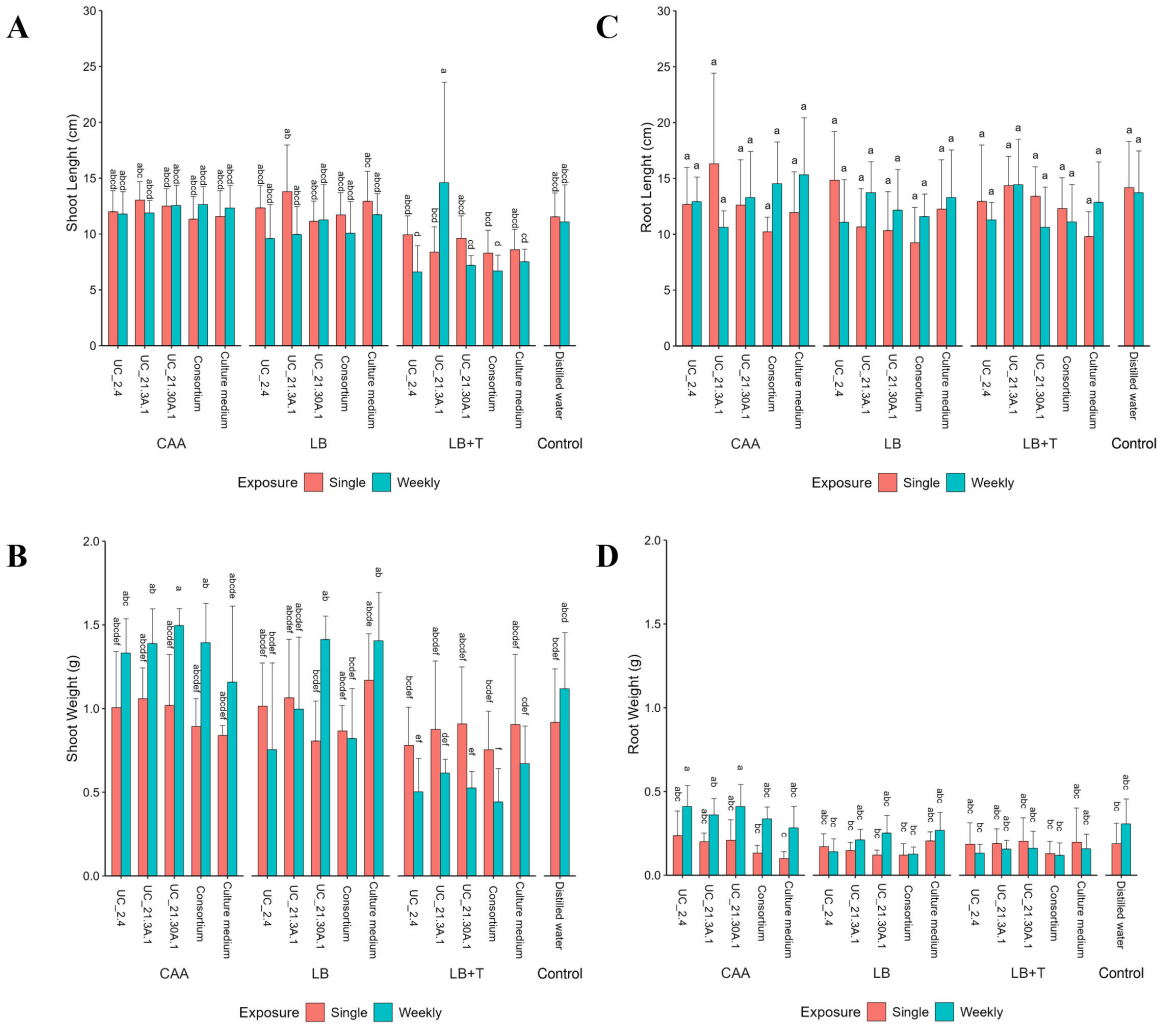
Effect of bacterial consortium strains on the growth of tomato plants (*S. lycopersicum* cv. Coração de Boi) in different media (CAA, LB, LB + Trp). Tomato plants were treated with individual or bacterial consortium strains, placed on pots filled with sand-peat soil and incubated for four weeks. Two treatment exposures were tested: single exposure (at time of potting) and weekly exposure (one time each week). Growth parameters measured included shoot length **(A)**, shoot weight **(B)**, root length **(C)**, and root weight **(D)**. Data are presented as averages from two independent bioassays. Three replicates per treatment. Standard deviations indicate variability among replicates. Different letters showing statistical differences (*p* < 0.05).

In pot assays, two independent bioassays were performed, and data were combined since they did not show statistical differences between them (**Supplementary Figure S5**). The length and weight of the shoots did not exhibit any significant differences between the several exposure conditions and culture media controls (**Figure 6**). Although slightly shorter shoot lengths were observed when the LB + Trp medium was used in bacterial growth, these differences did not reach statistical significance when compared to the other media. In addition, there were no statistically significant differences in root length under any of the experimental conditions. However, in terms of root weight, higher weights were recorded when the CAA medium was used, with higher values on weekly treatments. Specifically, statistically significant differences were detected in the context of weekly exposure with UC_2.4 growth in CAA, compared to equivalent conditions in the LB medium and the LB + Trp medium.

### Reduction of RKN infection in plants by bacterial consortium

**Figure 7:**
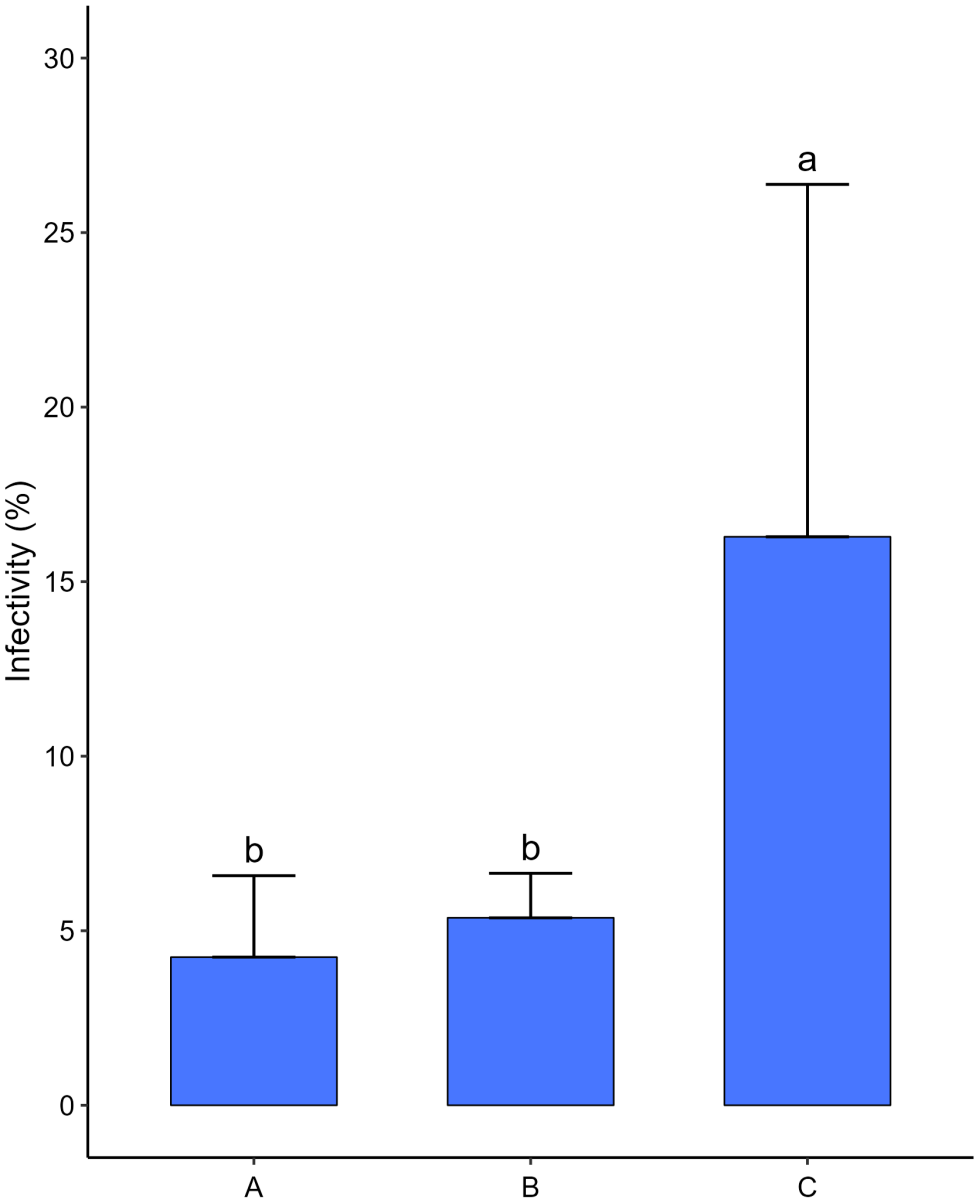
Effects of bacterial consortium strains on the infectivity of *Meloidogyne hapla* second stage juveniles (J2) in tomato plants (*S. lycopersicum* cv. Coração de Boi). **(A)** Bacterial consortium was added to pot followed by 300 J2 of *M. hapla*, 30 min interval. **(B)** Three hundred J2 of *M. hapla* was added to pot followed bacterial consortium, 30 min interval. **(C)** Three hundred J2 of *M hapla* was added to pot and SDW (control). Five replicates per treatment. Different letters showing statistical differences (*p* < 0.05).

The bacterial consortium significantly reduced the infectivity of *M. hapla*, which was in the control three times more on average than in the treatments (**Figure 7**). Considering the two treatments, with the bacterial consortium, there were no significant differences between them. However, the treatment, in which the bacterial consortium was added before the inoculation of the nematodes, was apparently more effective in reducing the infectivity of RKN.

## Discussion

PPN are an important issue in agriculture with tremendous economic impact since they cause a reduction in yield production and reduce the quality of the crops (Palomares-Rius et al., 2021). It is crucial to find new sustainable alternatives to overcome the use of pesticides to control agricultural pests, namely, PPN, and help to improve plant growth and preserve soil biodiversity. In our study, the consortium composed of *B. amyloliquefaciens* UC_2.4, *P. capeferrum* UC_21.3 A.1, and *P. capeferrum* UC_21.30 A.1 was able to act as nematicidal agents with 100% efficacy towards RKN but not against *C. elegans*, reduced the infectivity of RKN in plants by threefold, and showed some PGPB traits supported by genome mining and phenotypic assays *in vitro* and pot assays with plants. These findings support our hypothesis and demonstrate the effects of the bacterial consortium as a biological control agent in a more sustainable agricultural environment.

The bacteria in this study were previously isolated in natural potato-growing soils, and it was necessary to understand their role and biotechnological potential since the potato crops are also affected by PPN. By using phylogenomic analysis, the strains in the consortium belong to the species *B. amyloliquefaciens* and *P. capeferrum*. These species have been found in natural environments such as soil and water and are associated with plants (Höfte, 2021; Ngalimat et al., 2021). The use of genomic data improves our understanding of species classification, which is important to biological heritage preservation (Riesco and Trujillo, 2024).

The strain *B. amyloliquefaciens* UC_2.4 harbors in the genome genes encoding for NodB, nitrilase, and alpha-acetolactate decarboxylase, but not for the presence of genes encoding for periplasmic beta-glucosidase, catalase/peroxidase, aromatic L-amino acid decarboxylase, and pyruvate dehydrogenase that were present in strains *P. capeferrum* UC_21.3 A.1 and *P. capeferrum* UC_21.30 A.1. These genes have been reported as essential in plant–bacteria interactions, namely in the nitrogen cycle, degrading enzymes to colonize the plant tissues, and protecting the plants towards ROS (Chieb and Gachomo, 2023). These findings agree with previous studies since *B. amyloliquefaciens* and *P. capeferrum* have been reported as PGPB and biological control agents (Luo et al., 2022; O’Callaghan et al., 2022; Sheng et al., 2024).

Several key factors involved in enhancing plant growth are well-recognized and reviewed in Glick (2012). All strains showed siderophore production and catalase activity, key traits for promoting plant growth and fitness. Siderophores facilitate iron acquisition by plants from the soil, while catalase activity protects plants against reactive oxygen species (Chieb and Gachomo, 2023). In terms of nutrient solubilization, which is part of soil composition and important for plant growth, the *Pseudomonas* strains had zinc solubilization, and the *Bacillus* strain had phosphate solubilization. Strains produced IAA, with *P. capeferrum* UC_21.30 A.1 at the highest level, 4× more than strain UC_21.3 A.1, but the genes were not found in the genome’s sequences, which might be attributed to the incomplete genome sequence and the information missing that was not sequenced. Nevertheless, both strains show a high number of genes of the phenylalanine, tyrosine, and tryptophan biosynthesis pathway. The Trp-dependent IAA biosynthetic pathway can use the precursor tryptophan. Moreover, the *acdS* gene encoding for ACC deaminase was previously reported in *B. amyloliquefaciens* (Zafar-ul-Hye et al., 2019), but for *P. capeferrum* in our work, it is the first report of the presence of the gene for this species, and for both species, it agrees with the phenotypic activity observed. With a lower genome size, strain UC_21.3 A.1 showed more tRNAs and pseudogenes than strain UC_21.30 A.1. The ANI of 99.4% and DDH that ranged between 83.8% and 96.8% (depending on the method) support the idea that these are two strains of the same species with some differences in genomic content and phenotypic characteristics highlighted in this study.

As the plant health and fitness are affected by phytopathogenic fungi, the fungicidal activity of these bacterial strains towards two fungi was evaluated. Our study found that only the *Pseudomonas* or *Bacillus* strains exhibited activity against *B. cinerea* and *F. oxysporum*, respectively, in agreement with the literature (Balthazar et al., 2022).

In the test of biological control agents, performed towards *M. hapla* and *M. incognita*, all bacterial strains of the consortium displayed 100% nematicidal activity but were unable to significantly affect *C. elegans*, indicating a highly targeted action mechanism. This specificity is crucial since they can be used without affecting other non-target organisms, namely, beneficial soil nematodes, which play essential roles in soil health and ecosystem function (Neher, 2001). The mechanism involved in nematicidal activity was not the objective of this study, but the bacterial strains belonging to *B. amyloliquefaciens* and *P. capeferrum* are known to produce secondary metabolites, including lipopeptides such as surfactin, fengycin, and iturin; hydrogen cyanide; phenazines; and pyrrolnitrin that might be involved as key nematicide elements (Chen et al., 2007; Kenawy et al., 2019; Vasantha-Srinivasan et al., 2025; Wang et al., 2024). The genes involved in the production of surfactin, fengycin, and metallophores (azotobactin D, bacillibactin) were found in our strains. They might be involved in the management mechanism of PPN. Altogether, the complementary activity among these strains, demonstrated by genetic data and phenotypic assays, supports our establishment of this bacterial consortium.

Antibiotic susceptibility varies according to species and is strain dependent. When comparing our strains, *B. amyloliquefaciens* UC_2.4 showed more susceptibility to the antibiotics compared with the other two *Pseudomonas* strains, especially to erythromycin, which agrees with Blair et al. (2015) and Saha et al. (2008). *P. capeferrum* UC_21.3 A.1 and *P. capeferrum* UC_21.30 A.1 showed similar antibiotic reactions except for UC_21.30 A.1, which showed resistance to meropenem and differences in zone diameters regarding the neomycin test. Even though we found other differences in terms of antibiotic assays, we could not conclude whether they are susceptible or resistant since these strains were not classified by EUCAST for these antibiotics. This information is relevant not only for the characterization of the bacterial strains but also to the agrochemicals industry to understand the potential risk of use of some pesticides or antibiotics towards the environment to control pests that, on the other hand, also kill beneficial organisms for plants and, in our case, act as biological control agents. On the other hand, antibiotic resistance of bacterial strains might be identified as a pathogenicity factor because they could be spread by horizontal gene transfer and directly affect the soil biodiversity (Islam et al., 2024).

The market for commercial bacterial formulations is growing, and the most effective and popular ones are based on strains of the genera *Bacillus*, *Pseudomonas*, and *Serratia*. Youssef et al. (2017) revealed that *B. pumilis* and a bacterial complex, which included *Serratia* sp., *Pseudomonas* sp., *Azotobacter* sp., *B. circulans* and *B. thuringiensis* increased the length of the shoot, fresh and dry weight of the shoot, and sugar beet root (tuber) weight in greenhouse conditions. We tested our bacterial strains by growing them in LB medium supplemented with tryptophan (LB + TRP), since tryptophan promotes IAA production, alongside standard control (LB) and CAA medium, which, based on our previous research, is known to support the production of nematicidal compounds (Proença et al., 2019a, b). In this study, the consortium demonstrated some positive effects on plant growth, but only in terms of root weight. The discrepancy between an increase in weight of the roots in treatment with CAA medium did not correspond to an increase of the root length parameter, which might be due to an increase in lateral root formation or increased root thickness, which contributes to biomass but not to root length (Hernández-Amador et al., 2024). Effects on the aerial part were not observed, which may be attributed to several factors, such as the use of small pots, sterilized soil devoid of other microorganisms to interact with, and tightly controlled abiotic conditions. Further studies at the field level are necessary to evaluate the effect(s) of this consortium in real abiotic and biotic conditions.

As already mentioned, bacteria and their metabolites can affect both plant and invertebrate communities around the rhizosphere (Berg et al., 2017). Indirectly, bacteria can induce systemic resistance of the plants (Raymaekers et al., 2020). The most interesting and studied are bacterial strain members of the families *Bacillaceae* and *Pseudomonadaceae*, mainly on the control of species of RKN, because they occur in nature around the root system of plants (Dehghanian et al., 2020). Strains of these genera affect PPNs population density due to their efficacy as PGPB in the biological control of PPN and other pathogenic organisms (Raymaekers et al., 2020). In our work, the infectivity of *M. hapla* was affected by the bacterial consortium by reducing three times compared to the control. Our assay was performed by using 300 J2 nematodes, corresponding to 5 nematodes/g of soil, according to the recommended assay for this size (Collett et al., 2024). Topalović et al. (2020) showed that PGPB attached to J2 of *M. hapla* can induce plants’ immune system responses that inhibit RKN establishment.

Several works showed that some strains of *Pseudomonas* suppress the development of phytopathogenic fungi. Moreover, products formulated with *Bacillus* and *Pseudomonas* are more effective because they act as antagonists of PPN while also promoting plant growth and controlling plant pathogenic microorganisms. For instance, products like Poncho and VOTiVO, which are based on *B. firmus* have shown similar nematicidal activity when compared with the commercial pesticides Avicta for seed treatment and Aldicarb, which can be used as a nematicide (Migunova and Sasanelli, 2021).

Sustainable agriculture emphasizes the importance of green practices, and using bacterial consortia as biological control agents presents a promising strategy to reduce the use of chemicals as fertilizers and pesticides. The specificity of our unique bacterial consortium, composed of strains of *B. amyloliquefaciens* and *P. capeferrum* have shown effectiveness in controlling RKN without affecting *C. elegans*. This specificity, along with other PGPB traits, highlighted their potential as valuable tools in integrated pest management. Future research should focus on understanding the mechanisms of action, broader PPN species, and assessment of the long-term impacts of applying bacterial consortium in various agricultural practices.

## Data Availability

The datasets presented in this study can be found in online repositories. The names of the repository/repositories and accession number(s) can be found in the article/**Supplementary Material**.
