# Supplementary material for: Plant growth-promoting bacteria as biological control agents for sustainable agriculture: targeting root-knot nematodes
Source: Front Plant Sci. 2025 Aug 19;16:1567265. doi: 10.3389/fpls.2025.1567265 (PMC12401914; doi:10.3389/fpls.2025.1567265)
Supplement: Supplementary file 1 [file DataSheet1.pdf]

Plant Growth-Promoting Bacteria as Biological Control Agents for Sustainable Agriculture: Targeting Root-Knot Nematodes

Supplementary Material

Adriana S. Mata[1], Carlos Cruz[2], João Gaspar[1], Isabel Abrantes[2], Isabel Luci Conceição[2], Paula V. Morais[1], Diogo Neves Proença[1,3,*]

[1]University of Coimbra, Centre for Mechanical Engineering, Materials and Processes (CEMMPRE), Advanced Production and Intelligent Systems (ARISE), Department of Life Sciences, Coimbra, Portugal

[2]University of Coimbra, Centre for Functional Ecology - Science for People and the Planet (CFE), Associate Laboratory TERRA, Department of Life Sciences, Coimbra, Portugal

[3]MED – Mediterranean Institute for Agriculture, Environment and Development & CHANGE – Global Change and Sustainability Institute, Faculdade de Ciências e Tecnologia, Universidade do Algarve, Campus de Gambelas, Faro, Portugal

*Corresponding author:

Diogo Neves Proença

daproenca@ualg.pt

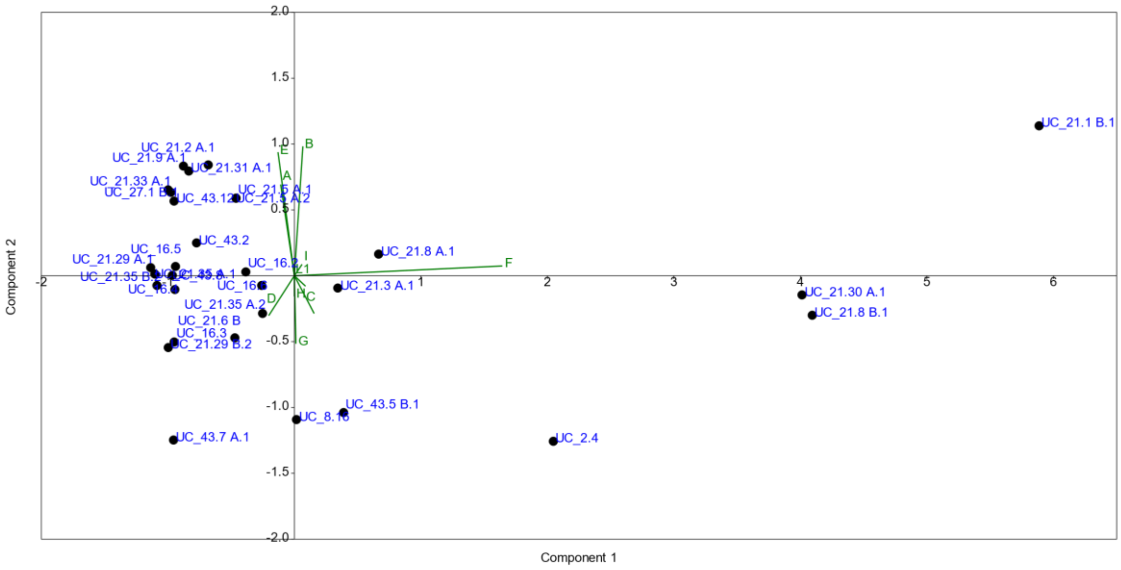

Figure S1. Principal component analysis (PCA) with the bacteria identified from potato-growing soil samples from this study. The first axis explains 70.0% and the second axis 9.3% of the variation. Plant growth-promoting bacteria (PGPB) characteristics of the strains, including phosphate solubilization (A), zinc solubilization (B), siderophores (C), proteases (D), lipases (Tween 20 | Tween 80) (E), IAA (µg/mL) (F), cellulolytic activity (G), chitinolytic activity (H), catalase (I), nematicidal activity (%) (J), susceptibility to antibiotics (K), fungicidal activity towards *Botrytis cinerea* Pars. (L1) or *Fusarium oxysporum* L21A50-1 (L2).

A

antiSMASH version 8.dev-0de8f3d7

Select genomic region: Overview 1.1 1.2 1.3 1.4 1.5 1.6 2.1 2.2 5.1 6.1 7.1 8.1 8.2 10.1 12.1 14.1 19.1 25.1

Identified secondary metabolite regions using strictness 'relaxed'

Region	Type	From	To	Confidence	Most similar known cluster	
Region 1.1	NRPS	1	38,294	Low	plipastatin	NRP
Region 1.2	terpene	66,161	88,044			
Region 1.3	T3PKS	151,019	192,119			
Region 1.4	terpene-precursor	269,442	290,491			
Region 1.5	transAT-PKS	351,166	457,348	High	difficidin	Polyketide
Region 1.6	terpene-precursor	480,680	501,570			
Region 2.1	transAT-PKS, T3PKS, NRPS-like, NRPS	193,821	303,923	High	bacillaene	Polyketide+NRP
Region 2.2	NRPS, betalactone, RiPP-like	369,528	457,506	High	fengycin	NRP
Region 5.1	lanthipeptide-class-ii	88,201	117,089			
Region 6.1	other	29,731	71,149	High	bacilysin	Other
Region 7.1	NRPS	146,237	204,395	High	surfactin	NRP:Lipopeptide
Region 8.1	terpene	31,023	51,763			
Region 8.2	PKS-like	134,564	175,808			
Region 10.1	HR-T2PKS	70,736	112,825			
Region 12.1	transAT-PKS	27,471	115,707	High	macrolactin H	Polyketide
Region 14.1	terpene-precursor	12,439	33,281			
Region 19.1	NRP-metallophore, NRPS	1	32,010	High	bacillibactin	NRP
Region 25.1	NRPS	1	9,922	Low	fengycin	NRP

B

antiSMASH version 8.dev-0de8f3d7

Select genomic region: Overview 1.1 1.2 2.1 2.2 4.1 5.1 7.1 13.1 15.1 17.1 18.1 19.1

Identified secondary metabolite regions using strictness 'relaxed'

Region	Type	From	To	Confidence	Most similar known cluster	
Region 1.1	NRP-metallophore, NRPS	40,370	120,968			
Region 1.2	NRP-metallophore, NRPS	389,282	456,250			
Region 2.1	NAGGN	1	9,960			
Region 2.2	hserlactone	437,370	457,963	Low	gamexpeptide A/gamexpeptide B/gamexpeptide E/luminmide B/luminmide D/luminmide E/luminmide F/luminmide G	NRP
Region 4.1	NRPS	1	68,773	High	putisolvin III/putisolvin VI/putisolvin V	NRP
Region 5.1	RiPP-like	165,428	176,264			
Region 7.1	ranthipeptide	78,080	99,472			
Region 13.1	terpene-precursor	15,322	36,290			
Region 15.1	redox-cofactor	59,443	81,602			
Region 17.1	ranthipeptide	48,411	69,841			
Region 18.1	RiPP-like	62,188	74,374			
Region 19.1	terpene-precursor	42,767	63,654			

C

antiSMASH version 8.dev-0de8f3d7

Select genomic region: Overview 1.1 1.2 2.1 2.2 5.1 6.1 10.1 12.1 13.1 17.1 18.1 20.1

Identified secondary metabolite regions using strictness 'relaxed'

Region	Type	From	To	Confidence	Most similar known cluster	
Region 1.1	ranthipeptide	80,301	101,693			
Region 1.2	triceptide	662,974	685,336			
Region 2.1	hserlactone	320,995	341,588	Low	gamexpeptide A/gamexpeptide B/gamexpeptide E/luminmide B/luminmide D/luminmide E/luminmide F/luminmide G	NRP
Region 2.2	terpene-precursor	508,173	529,141			
Region 5.1	RiPP-like	42,385	53,221			
Region 6.1	NRPS	35,101	115,230	High	putisolvin III/putisolvin VI/putisolvin V	NRP
Region 10.1	ranthipeptide	139,721	161,151			
Region 12.1	RiPP-like	109,426	121,612			
Region 13.1	NRP-metallophore, NRPS	1	35,502	Low	azotobactin D	NRP
Region 17.1	NAGGN	107,286	122,096			
Region 18.1	redox-cofactor	55,517	77,676			
Region 20.1	terpene-precursor	32,341	53,228			

Figure S2. Secondary biosynthetic gene clusters identified in strains *Bacillus amyloliquefaciens* UC_2.4 (A), *Pseudomonas capeferrum* UC_21.3 A.1 (B), and *P. capeferrum* UC_21.30 A.1 (C) with antiSMASH v8.

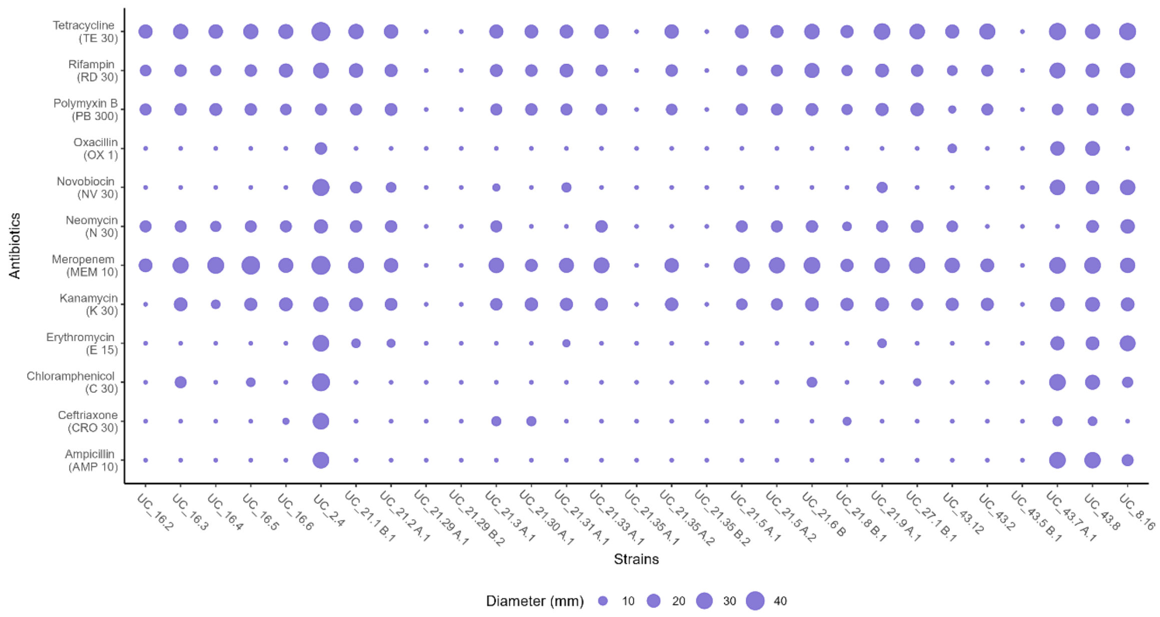

Figure S3. Antimicrobial susceptibility tests on Mueller-Hinton agar plates. Antibiotic susceptibility of 30 bacterial strains was assessed by measuring the diameter of the inhibition zones (mm) around antibiotic discs of 12 antibiotics.

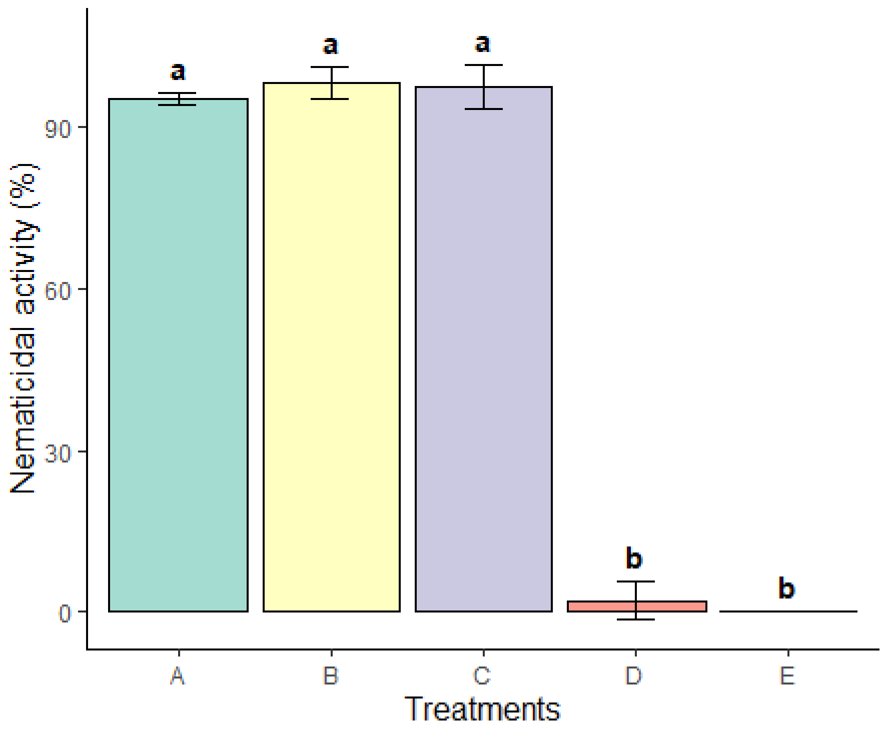

Figure S4. Nematicidal activity of bacterial supernatants towards *M. incognita*. Filtered bacterial supernatants (non-diluted) from the bacterial consortium strains *B. amyloliquefaciens* UC_2.4 (A), *P. capeferrum* UC_21.3 A.1 (B), and *P. capeferrum* UC_21.30 A.1 (C) grown in CAA medium were tested towards the plant-parasitic nematode *Meloidogyne incognita*. CAA medium (D) and distilled water (E) were used as controls. Five replicates per treatment. Standard deviations indicate variability among replicates. Different letters showing statistical differences ($p < 0.05$).

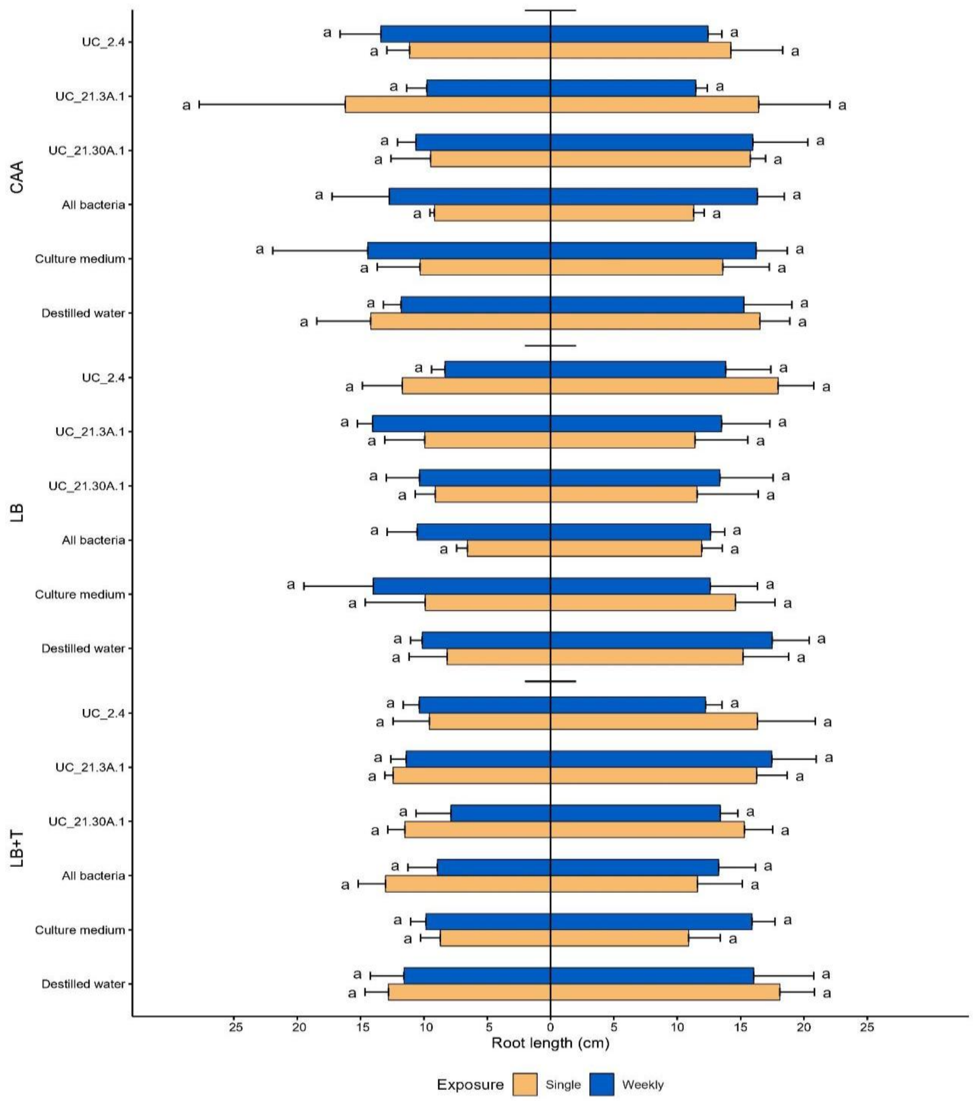

Figure S5. Pot assays with two independent bioassays. The root length of tomato plants was evaluated as single or weekly exposure conditions for bacterial strains grown in CAA, LB and LB+T media. No statistical differences were found between the two collection time points.

Table S1. Plant growth promoting bacterial (PGPB) characteristics of the bacterial strains, including phosphate solubilization (A), zinc solubilization (B), siderophores (C), proteases (D), lipases (Tween 20 | Tween 80) (E), IAA (µg/mL) (F), cellulolytic activity (G), chitinolytic activity (H), catalase (I), nematicidal activity (%) (J), susceptibility to antibiotics (K), fungicidal activity (0 h | 24 h) (L), *Botrytis cinerea* Pars. (L1), *Fusarium oxysporum* L21A50-1 (L2).

<table>
<tr><th rowspan="2">Strains</th><th rowspan="2">Sampling site GPS coordinates</th><th rowspan="2">Identification</th><th rowspan="2">A</th><th rowspan="2">B</th><th rowspan="2">C</th><th rowspan="2">D</th><th rowspan="2">E</th><th rowspan="2">F</th><th rowspan="2">G</th><th rowspan="2">H</th><th rowspan="2">I</th><th rowspan="2">J</th><th rowspan="2">K</th><th colspan="2">L</th></tr>
<tr><th>L1</th><th>L2</th></tr>
<tr><td>UC_2.4</td><td>40.304988,-8.453558</td><td>Bacillus amyloliquefaciens</td><td>-</td><td>-</td><td>+</td><td>+</td><td>- | -</td><td>341</td><td>+</td><td>-</td><td>+</td><td>100</td><td>Table S3</td><td>- | -</td><td>+ | +</td></tr>
<tr><td>UC_8.16</td><td>41.322030,-8.698454</td><td>Paenibacillus sp.</td><td>-</td><td>-</td><td>-</td><td>-</td><td>- | -</td><td>92</td><td>+</td><td>+</td><td>+</td><td>100</td><td>Table S3</td><td>- | -</td><td>- | -</td></tr>
<tr><td>UC_16.2</td><td>40.991503,-8.465817</td><td>Pseudomonas sp.</td><td>+</td><td>+</td><td>WP</td><td>+</td><td>- | -</td><td>62</td><td>-</td><td>-</td><td>+</td><td>100</td><td>Table S3</td><td>- | -</td><td>- | -</td></tr>
<tr><td>UC_16.3</td><td>40.991503,-8.465817</td><td>Pseudomonas sp.</td><td>+</td><td>-</td><td>-</td><td>+</td><td>- | -</td><td>14</td><td>-</td><td>-</td><td>+</td><td>100</td><td>Table S3</td><td>+ | +</td><td>- | -</td></tr>
<tr><td>UC_16.4</td><td>40.991503,-8.465817</td><td>Pseudomonas sp.</td><td>+</td><td>-</td><td>+</td><td>+</td><td>- | +</td><td>11</td><td>-</td><td>-</td><td>+</td><td>100</td><td>Table S3</td><td>- | -</td><td>- | -</td></tr>
<tr><td>UC_16.5</td><td>40.991503,-8.465817</td><td>Pseudomonas sp.</td><td>+</td><td>-</td><td>-</td><td>+</td><td>- | +</td><td>24</td><td>-</td><td>-</td><td>+</td><td>100</td><td>Table S3</td><td>- | -</td><td>- | -</td></tr>
<tr><td>UC_16.6</td><td>40.991503,-8.465817</td><td>Pseudomonas sp.</td><td>+</td><td>-</td><td>+</td><td>+</td><td>+</td><td>87</td><td>-</td><td>-</td><td>+</td><td>100</td><td>Table S3</td><td>- | -</td><td>- | -</td></tr>
<tr><td>UC_21.1 B.1</td><td>39.275148,-9.172316</td><td>Acinetobacter sp.</td><td>+</td><td>+</td><td>-</td><td>-</td><td>+</td><td>781</td><td>-</td><td>-</td><td>+</td><td>100</td><td>Table S3</td><td>- | -</td><td>- | -</td></tr>
<tr><td>UC_21.2 A.1</td><td>39.275148,-9.172316</td><td>Acinetobacter sp.</td><td>+</td><td>+</td><td>-</td><td>-</td><td>+</td><td>3</td><td>-</td><td>-</td><td>+</td><td>100</td><td>Table S3</td><td>- | -</td><td>- | -</td></tr>
<tr><td>UC_21.3 A.1</td><td>39.275148,-9.172316</td><td>Pseudomonas capeferrum</td><td>WP</td><td>+</td><td>+</td><td>-</td><td>- | -</td><td>116</td><td>-</td><td>-</td><td>+</td><td>100</td><td>Table S3</td><td>+ | +</td><td>- | -</td></tr>
<tr><td>UC_21.5 A.1</td><td>39.275148,-9.172316</td><td>Pseudomonas sp.</td><td>+</td><td>+</td><td>WP</td><td>+</td><td>+</td><td>62</td><td>-</td><td>-</td><td>+</td><td>100</td><td>Table S3</td><td>- | -</td><td>- | -</td></tr>
<tr><td>UC_21.5 A.2</td><td>39.275148,-9.172316</td><td>Pseudomonas sp.</td><td>+</td><td>+</td><td>WP</td><td>+</td><td>+</td><td>62</td><td>-</td><td>-</td><td>+</td><td>100</td><td>Table S3</td><td>- | -</td><td>- | -</td></tr>
<tr><td>UC_21.6 B</td><td>39.275148,-9.172316</td><td>Pseudomonas sp.</td><td>+</td><td>-</td><td>-</td><td>+</td><td>- | -</td><td>68</td><td>-</td><td>-</td><td>+</td><td>100</td><td>Table S3</td><td>- | -</td><td>- | -</td></tr>
<tr><td>UC_21.8 A.1</td><td>39.275148,-9.172316</td><td>Pseudomonas sp.</td><td>+</td><td>+</td><td>+</td><td>-</td><td>- | -</td><td>153</td><td>-</td><td>-</td><td>+</td><td>ND</td><td>Table S3</td><td>ND</td><td>ND</td></tr>
<tr><td>UC_21.8 B.1</td><td>39.275148,-9.172316</td><td>Pseudomonas sp.</td><td>+</td><td>-</td><td>+</td><td>-</td><td>- | -</td><td>549</td><td>-</td><td>-</td><td>+</td><td>100</td><td>Table S3</td><td>+ | +</td><td>- | -</td></tr>
<tr><td>UC_21.9 A.1</td><td>39.275148,-9.172316</td><td>Acinetobacter sp.</td><td>+</td><td>+</td><td>-</td><td>-</td><td>+</td><td>10</td><td>-</td><td>-</td><td>+</td><td>100</td><td>Table S3</td><td>- | -</td><td>- | -</td></tr>
<tr><td>UC_21.29 A.1</td><td>39.275148,-9.172316</td><td>Rudaibacter sp.</td><td>+</td><td>-</td><td>-</td><td>+</td><td>- | +</td><td>0</td><td>-</td><td>-</td><td>+</td><td>100</td><td>Table S3</td><td>- | -</td><td>- | -</td></tr>
<tr><td>UC_21.29 B.2</td><td>39.275148,-9.172316</td><td>Rudaibacter sp.</td><td>+</td><td>-</td><td>-</td><td>+</td><td>- | -</td><td>14</td><td>-</td><td>-</td><td>-</td><td>100</td><td>Table S3</td><td>- | -</td><td>- | -</td></tr>
<tr><td>UC_21.30 A.1</td><td>39.275148,-9.172316</td><td>Pseudomonas capeferrum</td><td>-</td><td>+</td><td>+</td><td>-</td><td>- | -</td><td>481</td><td>-</td><td>-</td><td>+</td><td>100</td><td>Table S3</td><td>+ | +</td><td>- | -</td></tr>
<tr><td>UC_21.31 A.1</td><td>39.275148,-9.172316</td><td>Acinetobacter sp.</td><td>+</td><td>+</td><td>-</td><td>-</td><td>+</td><td>14</td><td>-</td><td>-</td><td>+</td><td>100</td><td>Table S3</td><td>- | +</td><td>- | -</td></tr>
<tr><td>UC_21.33 A.1</td><td>39.275148,-9.172316</td><td>Pseudomonas sp.</td><td>+</td><td>+</td><td></td><td>+</td><td>+</td><td>14</td><td>-</td><td>-</td><td>+</td><td>100</td><td>Table S3</td><td>- | -</td><td>- | -</td></tr>
<tr><td>UC_21.35 A.1</td><td>39.275148,-9.172316</td><td>Rudaibacter sp.</td><td>+</td><td>-</td><td>-</td><td>+</td><td>+</td><td>7</td><td>-</td><td>-</td><td>-</td><td>99.6</td><td>Table S3</td><td>- | -</td><td>- | -</td></tr>
<tr><td>UC_21.35 A.2</td><td>39.275148,-9.172316</td><td>Pseudomonas sp.</td><td>+</td><td>-</td><td>-</td><td>-</td><td>- | -</td><td>71</td><td>-</td><td>-</td><td>+</td><td>100</td><td>Table S3</td><td>- | -</td><td>- | -</td></tr>
<tr><td>UC_21.35 B.2</td><td>39.275148,-9.172316</td><td>Rudaibacter sp.</td><td>+</td><td>-</td><td>WP</td><td>+</td><td>+</td><td>4</td><td>-</td><td>-</td><td>-</td><td>100</td><td>Table S3</td><td>- | -</td><td>- | -</td></tr>
<tr><td>UC_27.1 B.1</td><td>39.272188,-9.236129</td><td>Pseudomonas sp.</td><td>+</td><td>+</td><td>-</td><td>+</td><td>- | +</td><td>10</td><td>-</td><td>-</td><td>+</td><td>ND</td><td>Table S3</td><td>- | +</td><td>- | -</td></tr>
<tr><td>UC_43.2</td><td>40.235134,-8.453890</td><td>Pseudomonas sp.</td><td>+</td><td>+</td><td>-</td><td>-</td><td>- | -</td><td>12</td><td>-</td><td>-</td><td>+</td><td>ND</td><td>Table S3</td><td>- | +</td><td>- | -</td></tr>
<tr><td>UC_43.5 B.1</td><td>40.235134,-8.453890</td><td>Agromyces sp.</td><td>-</td><td>-</td><td>+</td><td>+</td><td>- | -</td><td>133</td><td>-</td><td>-</td><td>+</td><td>ND</td><td>Table S3</td><td>- | +</td><td>- | -</td></tr>
<tr><td>UC_43.7 A.1</td><td>40.235134,-8.453890</td><td>Bacillus sp.</td><td>-</td><td>-</td><td>-</td><td>+</td><td>- | -</td><td>0</td><td>+</td><td>-</td><td>+</td><td>ND</td><td>Table S3</td><td>+ | -</td><td>- | -</td></tr>
<tr><td>UC_43.8</td><td>40.235134,-8.453890</td><td>Bacillus sp.</td><td>+</td><td>-</td><td>WP</td><td>-</td><td>+</td><td>0</td><td>W+</td><td>-</td><td>+</td><td>ND</td><td>Table S3</td><td>- | -</td><td>- | -</td></tr>
<tr><td>UC_43.12</td><td>40.235134,-8.453890</td><td>Pseudomonas sp.</td><td>+</td><td>+</td><td>WP</td><td>+</td><td>+</td><td>12</td><td>-</td><td>-</td><td>+</td><td>ND</td><td>Table S3</td><td>- | -</td><td>- | -</td></tr>
</table>

Table S2. Genome comparison of *Pseudomonas capeferrum* UC_21.30 A.1 against *Pseudomonas capeferrum* UC_21.3 A.1.
(excel file)

Table S3. Genome comparison of *Pseudomonas capeferrum* UC_21.3 A.1 against *Pseudomonas capeferrum* strain UC_21.30 A.1.
(excel file)

Table S4. Genome mining of microbial consortium. (A) ACC deaminase (WP_009638971); (B) Lrp/AsnC family (WP_009638972); (C) Cellulase β-glucosidase (WP_009635056; WP_009636821); (D) Periplasmic β-glucosidase (WP_009638496); (E) Catalase (WP_009638086); (F) Catalase/peroxidase (WP_009636268); (G) Pectinase-pectinesterase B (WP_009638872); (H) Superoxide dismutase (WP_009635546); (I) Chitinase (WP_009638226); (J) nodB (WP_009639040, WP_009638391, WP_009639041); (K) nifH (nitrogenase) (AUG99286.1; H650_03210 K02588; pRL100162 K02588); (L) Aromatic-L-amino-acid decarboxylase (WP_009635138); (M) Aliphatic amidase AmiE (WP_009634947, WP_009639043); (N) Nitrilase (WP_009634944); (O) Aldehyde dehydrogenase (WP_009635369; WP_009635627; WP_009636235; WP_009637232; WP_009639021; WP_009639329); (P) Pyruvate dehydrogenase (WP_009634667); (Q) Acetolactate synthase (WP_009635236; WP_009634704; WP_009634705; WP_009637883; WP_009637884); (R) Alpha-acetolactate decarboxylase (WP_009635235); (S) 2,3-Butanediol dehydrogenase (WP_009635231); (T) Acyl-homoserine-lactone synthase (WP_009638744; WP_009635293); (U) LuxR family transcriptional regulator (WP_009638745; WP_009635292; WP_009637809; WP_009638756; WP_009635139; WP_037377293); (V) ᴅ-cysteine desulfhydrase (WP_009636121); (W) Myrosinase (NP_001302796.1; ALM58466.1); (X) Hydrocyanic acid (HCN).

Strains	Identification	Genome Ref	A	B	C	D	E	F	G	H	I	J	K	L	M	N	O	P	Q	R	S	T	U	V	W	X
UC_2.4	*Bacillus amyloliquefaciens*	JAWWVB010000000	-	+	-	-	+	-	-	+	-	+	-	-	+	+	+	-	+	+	-	-	+	-	-	-
UC_21.3 A.1	*Pseudomonas capeferrum*	JAWWVA010000000	-	+	-	+	+	+	-	+	-	-	-	+	+	-	+	+	+	-	-	-	+	-	-	-
UC_21.30 A.1	*Pseudomonas capeferrum*	JAWWUZ010000000	-	+	-	+	+	+	-	+	-	-	-	+	+	-	+	+	+	-	-	-	+	-	-	-

Table S5. Antibiotic resistance using EUCAST breakpoints tables Version 13.1.

<table>
<tr><th rowspan="2">Strains</th><th colspan="12">Susceptibility
(EUCAST breakpoints tables v 13.1)</th></tr>
<tr><th>Oxacillin[1]
(OX 1)</th><th>Ampicillin[1]
(AMP 10)</th><th>Ceftriaxone[1]
(CRO 30)</th><th>Kanamycin[1]
(K 30)</th><th>Neomycin[1]
(N 30)</th><th>Meropenem[1]
(MEM 10)</th><th>Erythromycin[1]
(E 15)</th><th>Novobiocin[1]
(NV 30)</th><th>Tetracycline[1]
(TE 30)</th><th>Rifampin[1]
(RD 30)</th><th>Chloramphenicol[1]
(C 30)</th><th>Polymyxin B[1]
(PB 300)</th></tr>
<tr><td>UC_2.4</td><td>16 |</td><td>30 |</td><td>30 |</td><td>25 |</td><td>21 |</td><td>40 | S</td><td>30 | S</td><td>32 |</td><td>40 |</td><td>27 |</td><td>36 |</td><td>15 |</td></tr>
<tr><td>UC_8.16</td><td>6 | R</td><td>15 |</td><td>6 | R</td><td>20 |</td><td>22 |</td><td>25 |</td><td>27 |</td><td>26 |</td><td>32 |</td><td>25 |</td><td>13 |</td><td>17 |</td></tr>
<tr><td>UC_16.2</td><td>6 | R</td><td>6 | R</td><td>6 | R</td><td>7 | R</td><td>15 |</td><td>20 |</td><td>6 | R</td><td>6 | R</td><td>21 |</td><td>14 |</td><td>6 | R</td><td>15 |</td></tr>
<tr><td>UC_16.3</td><td>6 | R</td><td>6 | R</td><td>6 | R</td><td>20 |</td><td>15 |</td><td>28 | S</td><td>6 | R</td><td>6 | R</td><td>25 |</td><td>16 |</td><td>15 |</td><td>16 |</td></tr>
<tr><td>UC_16.4</td><td>6 | R</td><td>6 | R</td><td>6 | R</td><td>10 |</td><td>13 |</td><td>31 | S</td><td>6 | R</td><td>6 | R</td><td>22 |</td><td>13 |</td><td>6 | R</td><td>17 |</td></tr>
<tr><td>UC_16.5</td><td>6 | R</td><td>6 | R</td><td>6 | R</td><td>18 |</td><td>15 |</td><td>38 | S</td><td>6 | R</td><td>6 | R</td><td>25 |</td><td>16 |</td><td>10 |</td><td>16 |</td></tr>
<tr><td>UC_16.6</td><td>6 | R</td><td>6 | R</td><td>7 |</td><td>20 |</td><td>15 |</td><td>24 | S</td><td>6 | R</td><td>6 | R</td><td>24 |</td><td>21 |</td><td>6 | R</td><td>14 |</td></tr>
<tr><td>UC_21.1 B.1</td><td>6 | R</td><td>6 | R</td><td>6 | R</td><td>20 |</td><td>16 |</td><td>28 | S</td><td>10 |</td><td>15 |</td><td>25 |</td><td>22 |</td><td>6 | R</td><td>15 |</td></tr>
<tr><td>UC_21.2 A.1</td><td>6 | R</td><td>6 | R</td><td>6 | R</td><td>17 |</td><td>16 |</td><td>22 | S</td><td>9 |</td><td>12 |</td><td>22 |</td><td>18 |</td><td>6 | R</td><td>17 |</td></tr>
<tr><td>UC_21.3 A.1</td><td>6 | R</td><td>6 | R</td><td>11 |</td><td>16 |</td><td>15 |</td><td>26 | S</td><td>6 | R</td><td>8 |</td><td>21 |</td><td>17 |</td><td>6 | R</td><td>15 |</td></tr>
<tr><td>UC_21.5 A.1</td><td>6 | R</td><td>6 | R</td><td>6 | R</td><td>14 |</td><td>15 |</td><td>28 | S</td><td>6 | R</td><td>6 | R</td><td>20 |</td><td>13 |</td><td>6 | R</td><td>15 |</td></tr>
<tr><td>UC_21.5 A.2</td><td>6 | R</td><td>6 | R</td><td>6 | R</td><td>15 |</td><td>15 |</td><td>28 | S</td><td>6 | R</td><td>6 | R</td><td>18 |</td><td>15 |</td><td>6 | R</td><td>15 |</td></tr>
<tr><td>UC_21.6 B</td><td>6 | R</td><td>6 | R</td><td>6 | R</td><td>20 |</td><td>16 |</td><td>38 | S</td><td>6 | R</td><td>6 | R</td><td>26 |</td><td>24 |</td><td>12 |</td><td>17 |</td></tr>
<tr><td>UC_21.8 B.1</td><td>6 | R</td><td>6 | R</td><td>9 |</td><td>18 |</td><td>10 |</td><td>18 |</td><td>6 | R</td><td>6 | R</td><td>18 |</td><td>13 |</td><td>6 | R</td><td>13 |</td></tr>
<tr><td>UC_21.9 A.1</td><td>6 | R</td><td>6 | R</td><td>6 | R</td><td>20 |</td><td>15 |</td><td>26 | S</td><td>10 |</td><td>13 |</td><td>30 | S</td><td>20 |</td><td>6 | R</td><td>18 |</td></tr>
<tr><td>UC_21.29 A.1</td><td>6 | R</td><td>6 | R</td><td>6 | R</td><td>6 | R</td><td>6 | R</td><td>6 | R</td><td>6 | R</td><td>6 | R</td><td>6 | R</td><td>6 | R</td><td>6 | R</td><td>6 | R</td></tr>
<tr><td>UC_21.29 B.2</td><td>6 | R</td><td>6 | R</td><td>6 | R</td><td>6 | R</td><td>6 | R</td><td>6 | R</td><td>6 | R</td><td>6 | R</td><td>6 | R</td><td>6 | R</td><td>6 | R</td><td>6 | R</td></tr>
<tr><td>UC_21.30 A.1</td><td>6 | R</td><td>6 | R</td><td>11 |</td><td>19 |</td><td>6 | R</td><td>17 | R</td><td>6 | R</td><td>6 | R</td><td>20 |</td><td>16 |</td><td>6 | R</td><td>16 |</td></tr>
<tr><td>UC_21.31 A.1</td><td>6 | R</td><td>6 | R</td><td>6 | R</td><td>18 |</td><td>6 | R</td><td>24 | S</td><td>8 |</td><td>11 |</td><td>20 | S</td><td>20 |</td><td>6 | R</td><td>15 |</td></tr>
<tr><td>UC_21.33 A.1</td><td>6 | R</td><td>6 | R</td><td>6 | R</td><td>18 |</td><td>16 |</td><td>27 | S</td><td>6 | R</td><td>6 | R</td><td>22 |</td><td>15 |</td><td>6 | R</td><td>14 |</td></tr>
<tr><td>UC_21.35 A.1</td><td>6 | R</td><td>6 | R</td><td>6 | R</td><td>6 | R</td><td>6 | R</td><td>6 | R</td><td>6 | R</td><td>6 | R</td><td>6 | R</td><td>6 | R</td><td>6 | R</td><td>6 | R</td></tr>
<tr><td>UC_21.35 A.2</td><td>6 | R</td><td>6 | R</td><td>6 | R</td><td>19 |</td><td>6 | R</td><td>22 |</td><td>6 | R</td><td>6 | R</td><td>22 |</td><td>16 |</td><td>6 | R</td><td>14 |</td></tr>
<tr><td>UC_21.35 B.2</td><td>6 | R</td><td>6 | R</td><td>6 | R</td><td>6 | R</td><td>6 | R</td><td>6 | R</td><td>6 | R</td><td>6 | R</td><td>6 | R</td><td>6 | R</td><td>6 | R</td><td>6 | R</td></tr>
<tr><td>UC_27.1 B.1</td><td>6 | R</td><td>6 | R</td><td>6 | R</td><td>15 |</td><td>17 |</td><td>29 | S</td><td>6 | R</td><td>6 | R</td><td>26 |</td><td>16 |</td><td>8 |</td><td>19 |</td></tr>
<tr><td>UC_43.2</td><td>6 | R</td><td>6 | R</td><td>6 | R</td><td>18 |</td><td>6 | R</td><td>20 |</td><td>6 | R</td><td>6 | R</td><td>28 |</td><td>15 |</td><td>6 | R</td><td>15 |</td></tr>
<tr><td>UC_43.5 B.1</td><td>6 | R</td><td>6 | R</td><td>6 | R</td><td>6 | R</td><td>6 | R</td><td>6 | R</td><td>6 | R</td><td>6 | R</td><td>6 | R</td><td>6 | R</td><td>6 | R</td><td>6 | R</td></tr>
<tr><td>UC_43.7 A.1</td><td>22 |</td><td>30 |</td><td>11</td><td>22 |</td><td>6 | R</td><td>30 | S</td><td>24 | S</td><td>26 |</td><td>32 |</td><td>27 |</td><td>30 |</td><td>14 |</td></tr>
<tr><td>UC_43.8</td><td>23 |</td><td>29 |</td><td>10</td><td>23 |</td><td>17 |</td><td>30 | S</td><td>24 | S</td><td>20 |</td><td>25 |</td><td>21 |</td><td>24 |</td><td>15 |</td></tr>
<tr><td>UC_43.12</td><td>10 |</td><td>6 | R</td><td>6 | R</td><td>18 |</td><td>14 |</td><td>25 | S</td><td>6 | R</td><td>6 | R</td><td>21 |</td><td>12 |</td><td>6 | R</td><td>8 |</td></tr>
</table>

[1]Results presented as zone diameter (mm) [includes 6 mm of the disk diameter] | susceptibility categories [Resistant (R); Susceptible, Increased Exposure (I); and Susceptible, standard dosing regimen (S)].
